# On the Classification of Bosonic and Fermionic One-Form Symmetries in $2+1$d and ’t Hooft Anomaly Matching

**DOI:** 10.1007/s00220-025-05494-0

**Published:** 2025-11-20

**Authors:** Mahesh Balasubramanian, Matthew Buican, Rajath Radhakrishnan

**Affiliations:** 1 CTP and Department of Physics and Astronomy, Queen Mary University of London, London, E1 4NS UK; 2 International Centre for Theoretical Physics, Strada Costiera 11, Trieste, 34151 Italy

## Abstract

Motivated by the fundamental role that bosonic and fermionic symmetries play in physics, we study finite (non-invertible) one-form symmetries in $2+1$d consisting of topological lines with bosonic and fermionic self-statistics. We refer to these lines as Bose–Fermi–Braided (BFB) symmetries and argue that they can be classified. Unlike the case of generic anyonic lines, BFB symmetries are closely related to groups. In particular, when BFB lines are non-invertible, they are non-intrinsically non-invertible. Moreover, BFB symmetries are, in a categorical sense, weakly group theoretical. Using this understanding, we study invariants of renormalization group flows involving non-topological QFTs with BFB symmetry.

## Introduction

Bosonic and fermionic groups and algebras play foundational roles in constraining QFTs in various dimensions (e.g., see the classic results of [1–3]). Recently, considerable attention has been paid to a vast generalization of the notion of symmetry in which one replaces groups with categories of topological defects implementing symmetries that are generally non-invertible (e.g., see [4–6] for recent reviews).

In the context of $2+1$d QFTs, quasi-particles and their braided worldlines often obey an interesting and relatively “wild” anyonic, or fractional, generalization of Bose/Fermi statistics characterized by complex phases and, more generally, unitary matrices [7, 8]. When they are topological, these $2+1$d anyonic lines generate one-form symmetries that are typically non-invertible (e.g., as in the case of Wilson lines in generic non-Abelian Chern-Simons theories).^1^

Given this picture, a natural first question is to try to classify the finite one-form symmetries arising from topological lines in $2+1$d that are bosonic and fermionic (here we define bosonic and fermionic lines to have self-statistics, or topological spin, $\theta =1$ and $\theta =-1$ respectively).^2^ One might imagine that, unlike more generic one-form symmetries, these one-form symmetries are closely related to groups. Indeed, we will show this intuition is correct by demonstrating that:

*Any symmetry category,* $\mathcal {B}$, *consisting solely of bosonic and fermionic lines is related to groups in (at least) two ways:* **(1)** $\mathcal {B}$ *is weakly group theoretical (in the categorical sense* [10]) *and* **(2)** *If* $\mathcal {B}$ *is non-invertible, it is non-intrinsically non-invertible.*^3^

Depending on the context, we will refer to such $\mathcal {B}$ symmetries as Bose–Fermi–Braided (BFB) symmetries or BFB categories. Roughly speaking, the properties described in the italics above mean that any BFB category can, by topological manipulations, be related to invertible objects forming a group.^4^ Note that general anyonic one-form symmetries are neither weakly group theoretical nor intrinsically non-invertible (a particularly simple example is the Fibonacci category arising from the two lines in $(G_2)_1$ Chern-Simons theory).

The relative “tameness” of BFB categories allows us to get a handle on these symmetries and argue that:

- All BFB symmetries can be classified (with the classification of BFB categories lacking a transparent fermion being particularly explicit) and realized. It is rare that infinite families of (non-Abelian) symmetry categories can be classified (exceptions include categories with trivial braiding, which are simple examples of BFB categories, and “metaplectic” modular categories [15]^5^). We put this classification to work by deriving invariants of renormalization group (RG) flows involving QFTs that have BFB symmetries. We interpret these invariants as relatives of the spectator sectors ’t Hooft used in his original anomaly matching arguments [16].
- An important subclass of BFB symmetries are full-fledged (spin) TQFTs. We can connect any such BFB (spin) TQFT with a non-topological UV completion. In other words, we can construct explicit RG flows that result in any BFB (spin) TQFT as a gapped IR phase. For general topological phases, such an explicit connection seems out of reach, but we hope that our results can serve as simple stepping stones for connections between classes of more general topological phases and the RG flow.

The plan of this paper is as follows. In the next section we introduce further details of BFB symmetries and focus on the case that $\mathcal {B}$ corresponds to a (spin) TQFT. Then, in Sect. 2.1, we provide some simple UV completions of these TQFTs via circular quivers and decoupled product QCD theories. We move on to more general $\mathcal {B}$ in Sect. 2.2 and give a proof of the italicized statement above. Then, in Sect. 2.3, we give a concrete characterization of general BFB categories lacking transparent fermions. In Sect. 3 we consider the RG consequences of our analysis, and we conclude with a discussion of open problems. Except in Appendix A, we assume that the braided fusion category of line operators is unitary.

## Bose–Fermi–Braided (BFB) Categories

In this section, we characterize one-form symmetries consisting of bosons and fermions. Note that we *do not* assume these symmetries are invertible. As described in the introduction, we interchangeably refer to such symmetries as BFB symmetries or BFB categories depending on the context. They consist of line operators with bosonic or fermionic self-statistics that are closed under fusion and have a notion of braiding. In a more mathematical language, BFB symmetries are “premodular” categories with real twists (e.g., see [10] for a definition of a premodular category). Moreover, since we are interested in unitary QFTs, we assume that the braided fusion category of line operators is unitary (see Appendix A for a relaxing of this condition).

Roughly speaking, we would like to classify collections of line operators that can have non-trivial mutual statistics but are not “genuinely” anyonic (in the perhaps misleading sense of not having fractional self-statistics; note that these lines are genuine line operators and are not attached to surfaces). Examples of such collections of line operators include Kitaev’s toric code modular tensor category (MTC) [17], which is one of the simplest examples of BFB topological order and will play an important role below.

To describe BFB symmetries, we begin with the modular data^6^

2.1 $$\begin{aligned} \theta (\ell _i)\in \left\{ \pm 1\right\} ,\ \ \ S_{\ell _i\ell _j}=\sum _{\ell _k}N_{\ell _i\bar{\ell }_j}^{\ell _k}{\theta (\ell _k)\over \theta (\ell _i)\theta (\ell _j)}d_{\ell _k}, \end{aligned}$$

which characterize the self-statistics and mutual-statistics of the lines, $\ell _{i,j}\in \mathcal {B}$, of the BFB category, $\mathcal {B}$, respectively. In writing the modular *S*-matrix, we sum over simple lines, $\ell _k\in \mathcal {B}$, and weight the sum by the non-negative integer fusion coefficients

2.2 $$\begin{aligned} \ell _i\times \ell _j=\sum _{\ell _k}N_{\ell _i\ell _j}^{\ell _k}\ell _k, \end{aligned}$$

and real quantum dimensions, $d_{\ell _k}\in \mathbb {R}_{\ge 1}$.^7^ Note that the quantum dimensions satisfy the fusion rules

2.4 $$\begin{aligned} d_{\ell _i}\cdot d_{\ell _j}=\sum _{\ell _k}N_{\ell _i\ell _j}^{\ell _k}d_{\ell _k}~. \end{aligned}$$

Therefore, non-invertible lines (i.e., those satisfying $\ell \times \bar{\ell }=1+\cdots$, with non-trivial lines in the ellipses) have $d_{\ell }>1$, while invertible lines have $d_{\ell }=1$.

**Fig. 1:**
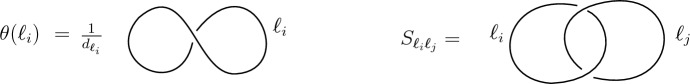
BFB categories have $\mathbb {R}$-valued modular data

One obvious fact that follows from (2.1) is that *S* is real. As a result, in BFB categories, both the self-statistics and mutual-statistics of lines are governed by real numbers (see Fig. 1). Another trivial fact following from this discussion is that all lines in a BFB category *with invertible* *S* are self-dual

2.5 $$\begin{aligned} \ell _i\times \ell _i\ni 1, \end{aligned}$$

where “1” denotes the trivial line. To appreciate this point, consider an invertible and real *S*-matrix. Unitarity of the S-matrix implies

2.6 $$\begin{aligned} \sum _{\ell _j} S_{\ell _i\ell _j}S_{\ell _j\ell _k} = \delta _{\ell _i\ell _k} D^2~. \end{aligned}$$

Now, using the property that $D^2\,S^{-1}_{\ell _j\ell _k}=S_{\ell _j\bar{\ell }_k}$ [17], we see that $\ell _k =\bar{\ell }_k$ for all $\ell _k \in \mathcal {B}$. In more general BFB categories, (2.5) does not necessarily hold (e.g., consider $\mathcal {B}\cong {\textrm{Rep}}(G)$ for a non-ambivalent group, *G*).

We can contemplate two extremes for the *S* matrix of $\mathcal {B}$, namely that it is completely degenerate or that it is invertible. In the case that it is degenerate, a theorem of Deligne [18] guarantees that the lines form the representation category of some finite (super) group, $\mathcal {B}\cong {\textrm{Rep}}(G_z)$ (see also the work of Doplicher and Roberts [19, 20]). We will describe such cases in more detail below. We should think of such a $\mathcal {B}$ as corresponding to a sub-sector of a non-topological QFT, $\mathcal {Q}$, rather than as characterizing a topological phase of matter. For example, in one of its guises, $\mathcal {B}\cong {\textrm{Rep}}(\mathbb {Z}_2)$ appears as the one-form symmetry of pure $2+1$d *SU*(2) Yang-Mills (YM) theory [9].

When *S* is non-degenerate, $\mathcal {B}$ describes a non-spin TQFT, i.e. a TQFT that does not depend on a spin structure (for convenience, we will drop the “non-spin” modifier). In the language of category theory, $\mathcal {B}$ corresponds (as a 1-category) to an MTC, $\mathcal {B}\cong \mathcal {M}$.

To get an idea of what is possible in the non-degenerate case, let us first consider the case in which all non-trivial lines, $\ell _i\in \mathcal {B}$ (i.e., $\ell _i\ne 1$), are fermions. An example of such an MTC is the “3-fermion” MTC, $F_2$ (using the notation of [21]), described by the following topological spins and *S* matrix^8^

2.7 $$\begin{aligned} \theta (1)=1 ,\ \theta (\psi _1)=\theta (\psi _2)=\theta (\psi _3)=-1 ,\ \ \ S=\begin{pmatrix} 1 & \quad 1 & \quad 1& \quad 1\\ 1 & \quad 1 & \quad -1& \quad -1\\ 1 & \quad -1 & \quad 1& \quad -1\\ 1 & \quad -1 & \quad -1& \quad 1\\ \end{pmatrix}~. \end{aligned}$$

This theory consists of invertible/Abelian lines with $\mathbb {Z}_2\times \mathbb {Z}_2$ fusion rules. In fact, from the list of prime Abelian MTCs in [21],^9^ it is easy to see that this is the only Abelian theory whose non-trivial lines are all fermions. In the language of Chern–Simons (CS) theory, we can obtain such an MTC from $\textrm{Spin}(N)_1$ with $N=8\ \textrm{mod}\ 16$

2.9 $$\begin{aligned} \textrm{Spin}(N)_1\ \leftrightarrow \ \mathrm{3-fermion\ MTC}\cong F_2\ \textrm{MTC} , \ \ \ N=8\ \textrm{mod}\ 16~. \end{aligned}$$

Next let us consider theories in which all non-trivial lines are fermions and we also allow for non-Abelian fusion. Using the general expression for the *S* matrix in (2.1), and requiring the *S* matrix to be invertible, it is easy to see that the only possibility has three simple lines with

2.10 $$\begin{aligned} S=\begin{pmatrix} 1 & \quad 1 & \quad \sqrt{2}\\ 1 & \quad 1 & \quad -\sqrt{2}\\ \sqrt{2} & \quad -\sqrt{2} & \quad 0\\ \end{pmatrix}~. \end{aligned}$$

This result follows from the fact that the combination of topological spins, $\theta (\ell _k)/\theta (\ell _i)\theta (\ell _j)$, entering the expression for the *S* matrix in (2.1) is equal to minus one for $\ell _{i,j,k}\ne 1$ and one otherwise. However, the above theory is inconsistent. Indeed, it has Ising fusion rules: $\sigma \times \sigma =1+\epsilon$, with 1 and $\epsilon$ invertible (where we write *S* in (2.10) in the basis $\left\{ 1,\epsilon ,\sigma \right\}$). The issue is that the non-invertible (Kramers–Wannier duality) line, $\sigma$, cannot be fermionic in such a theory but rather must have anyonic self-statistics (given by a primitive sixteenth root of unity).^10^

As a result, we arrive at the following simple theorem:

### Theorem 1

The only MTC whose non-trivial simple lines are fermions is the 3-fermion (a.k.a. $F_2$) MTC. One realization of this MTC is via the Wilson lines of any $\textrm{Spin}(N)_1$ CS TQFT with $N=8$ mod 16.

What can we say about the most general BFB TQFT? In this case, the simple argument involving the *S* matrix below (2.10) no longer works because the combination of topological spins that we use is less constrained. To avoid complications from the topological spins, we would like to study an observable built from modular data that is quadratic in spins, so that the spin dependence drops out for BFB theories. Another hint regarding which observable to use arises from the fact that all lines in our theory are in fact self-dual and therefore have a non-vanishing Frobenius-Schur (FS) indicator, $\nu _2(\ell _i)=\pm 1$. The fact that the FS indicator has value $\pm 1$ arises from the fact that it can be understood as a $\mathbb {Z}_2$ action on the $a\times a\ni 1$ fusion space.^11^

More precisely, the FS indicator is defined as

**Fig. 2:**
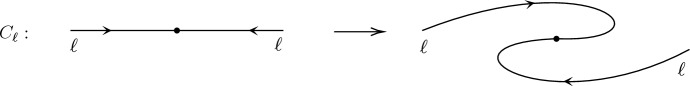
$C_\ell : V_{\ell \ell }^{1} \rightarrow V_{\ell \ell }^{1}$ acts on the fusion vertex by rotating it clockwise by an angle $\pi$

2.11 $$\begin{aligned} \nu _2(\ell ):= \text {Tr}(C_\ell ) , \end{aligned}$$

where $C_\ell$ is the action on the fusion space, $V_{\ell \ell }^1$, given in Fig. 2. From the pivotal property of the category (which follows from unitarity, see [17, Fig. 16]) rotating a fusion vertex by an angle $2\pi$ must be the identity operation. Therefore, $C_\ell$ is order two. It follows that the eigenvalues of $C_\ell$ are valued in $\pm 1$. In fact, since $V_{\ell \ell }^{1}$ is at most 1-dimensional, $C_\ell = \pm 1$, and $\nu _2(\ell )=\pm 1$.^12^

Since the FS indicator gives a gauge-invariant measure of the total angular momentum in an anyonic system [17], we expect to find an expression in terms of the modular data. Indeed, according to [26–28], we have

2.12 $$\begin{aligned} \nu _2(\ell _i)= \frac{1}{D^2} \sum _{\ell _{j},\ell _k\in \mathcal {B}} N_{\ell _j\ell _k}^{\ell _i} d_{\ell _j} d_{\ell _k} \bigg (\frac{\theta (\ell _j)}{\theta (\ell _k)} \bigg )^2~. \end{aligned}$$

From the expression in (2.12), it is then easy to see that since a BFB TQFT only has bosonic and fermionic spins

2.13 $$\begin{aligned}  &   \nu _2(\ell _i)= \frac{1}{D^2} \sum _{\ell _j,\ell _k\in \mathcal {B}} N_{\ell _j\ell _k}^{\ell _i} d_{\ell _j} d_{\ell _k} = \frac{1}{D^2} \sum _{\ell _j,\ell _k\in \mathcal {B}} \left( N_{\ell _i \ell _j}^{\ell _k} d_{\ell _k}\right) \nonumber \\  &   \quad d_{\ell _j} = \frac{1}{D^2} \left( \sum _{\ell _j\in \mathcal {B}} d_{\ell _j}^2\right) d_{\ell _i}=d_{\ell _i}~. \end{aligned}$$

In the third equality, we have used the fact that quantum dimensions satisfy fusion rules as in (2.4). Since the FS indicator is plus or minus one, we learn that $d_{\ell _i}=\pm 1$, but, in a unitary theory (which we assume throughout the main text), $d_{\ell _i}=1$, and we have

2.14 $$\begin{aligned} d_{\ell _i}=1 ,\ \ \ \forall \ell _i\in \mathcal {B}~. \end{aligned}$$

In other words, we see that all BFB TQFTs consist of Abelian / invertible lines! In fact, this statement was already derived in [29, 30] using essentially the same arguments.

From the classification of Abelian MTCs in [21], it is easy to see that the most general BFB MTC we can write down involves a stacking of MTCs corresponding to the 3-fermion MTC we encountered around (2.9) and the $E_2$ MTC in the nomenclature of [21]. This latter MTC can be equivalently realized by (among other quantum systems) Kitaev’s toric code at low energies, the untwisted $\mathbb {Z}_2$ Dijkgraaf–Witten (DW) theory, or $\textrm{Spin}(N)_1$ CS theory with $N=0$ mod 16. In other words, we have

2.15 $$\begin{aligned} \textrm{Spin}(N)_1 ,\ \mathbb {Z}_2\ \mathrm{untwisted\ DW\ theory}\ \leftrightarrow \ \mathrm{toric\ code\ MTC}\cong E_2\ \textrm{MTC} , \ \ \ N=0\ \textrm{mod}\ 16 , \end{aligned}$$

and, we arrive at the following theorem:

### Theorem 2

The most general BFB TQFT corresponds to the following MTC (see also [30])

2.16 $$\begin{aligned} \mathcal {M}\cong (E_2)^{\boxtimes n}\boxtimes (F_2)^{\boxtimes m} ,\ \ \ (X)^{\boxtimes p}:=\overbrace{X\boxtimes X\boxtimes \cdots \boxtimes X}^\mathrm{p\ times}~. \end{aligned}$$

In fact, using the equivalence $E_2\boxtimes E_2\cong F_2\boxtimes F_2$, we can simplify $\mathcal {M}$ as follows

2.17 $$\begin{aligned} \mathcal {M}\cong (E_2)^{\boxtimes n}\boxtimes (F_2)^{\boxtimes m} ,\ \ \ n=0,1 ,\ \ \ m\ge 0~. \end{aligned}$$

At the level of CS theories, such an MTC can be realized by, for example, stacking *n* $\textrm{Spin}(N)_1$ CS theories ($N=0$ mod 16) with *m* $\textrm{Spin}(N')_1$ CS theories ($N'=8$ mod 16).

Let us now consider BFB symmetries corresponding to a degenerate *S* matrix. Here it is useful to use a more general expression for the FS indicator in premodular categories [27, 28]

2.18 $$\begin{aligned} \nu _2(\ell _i)= &   \frac{1}{D^2} \sum _{\ell _{j},\ell _k\in \mathcal {B}} N_{\ell _j\ell _k}^{\ell _i} d_{\ell _j} d_{\ell _k} \bigg (\frac{\theta (\ell _j)}{\theta (\ell _k)} \bigg )^2-\theta (\ell _i)\sum _{\ell \in \mathcal {Z}_M(\mathcal {B}), \ell \ne 1}\textrm{Tr}(R_{\ell _i\ell _i}^{\ell })\cdot d_{\ell }\nonumber \\= &   d_{\ell _i}-\theta (\ell _i)\sum _{\ell \in \mathcal {Z}_M(\mathcal {B}), \ell \ne 1}\textrm{Tr}(R_{\ell _i\ell _i}^{\ell })\cdot d_{\ell } , \end{aligned}$$

where $\mathcal {Z}_M(\mathcal {B})$ is the so-called “Müger center” of $\mathcal {B}$ [31], and $R_{\ell _i\ell _j}^{\ell _k}$ is the braiding matrix. Physically $\mathcal {Z}_M(\mathcal {B})$ is the set of line operators in $\mathcal {B}$ that braid trivially with all lines in $\mathcal {B}$ (i.e., the set of “transparent” lines). It forms a fusion subcategory of $\mathcal {B}$ [31, Lemma 2.8]. In the second equality we have used logic similar to that around (2.13). The formula (2.18) for the FS indicator can be applied to both self-dual and non-self-dual lines. This statement holds because the argument leading to this formula in [28] can be repeated for non-self dual lines (where, as in footnote 12, we take $\nu _2(\ell ):=0$ when $\ell \ne \bar{\ell }$).

We can simplify (2.18) further. Indeed, we know from Deligne’s theorem that, for $\ell$ to be transparent, it should be a boson or a fermion (i.e., it cannot have anyonic self statistics). However, a transparent fermion, $\ell =\psi$, cannot appear in $\ell _i\times \ell _i$. Indeed, otherwise $\psi \times \bar{\ell }_i\ni \ell _i$, and $\psi$ would braid non-trivially with $\ell _i$. Therefore, we arrive at

2.19 $$\begin{aligned} \nu _2(\ell _i)=d_{\ell _i}-\theta (\ell _i)\sum _{\ell \in \mathcal {Z}^\textrm{bos}_M(\mathcal {B}), \ell \ne 1}\textrm{Tr}(R_{\ell _i\ell _i}^{\ell })\cdot d_{\ell } , \end{aligned}$$

where the “bos” superscript in the summation refers to the fact that only transparent bosons contribute.

We can motivate the formula in (2.19) as follows. At a basic level, the correction term arising from the Müger center is required in order to reproduce what we already know: in the case of symmetric $\mathcal {B}$ governed by Deligne’s theorem, $\mathcal {Z}_M(\mathcal {B})\cong \mathcal {B}$, we can have non-Abelian lines, $\ell _i\in \mathcal {B}\cong \textrm{Rep}(G)$, when *G* is non-Abelian (i.e., $d_{\ell _i}=\textrm{dim}(\pi )>1$ for a non-Abelian irrep $\pi \in \textrm{Rep}(G)$). Moreover, we can also have non-self-dual lines (when $\mathcal {B}$ is symmetric, such lines occur whenever *G* is not ambivalent) and lines with negative FS indicator (this situation occurs for lines labeled by pseudo-real representations of *G*).

To give further motivation for the correction term in (2.19), note that contributions from $\ell \in \mathcal {Z}_M^\textrm{bos}(\mathcal {B})$ satisfying $\ell \in \ell _i\times \ell _i$ turn out to be crucial because these are precisely the bosonic $\ell$ that satisfy $\ell \times \bar{\ell }_i\ni \ell _i$. When $\ell _i$ is self-dual, condensing $\ell$ can produce Abelian lines. Moreover, when $\ell _i\ne \bar{\ell }_i$, condensing $\ell$ can produce self-dual lines. Since condensing $\mathcal {Z}_M^\textrm{bos}(\mathcal {B})$ gives a (possibly trivial) TQFT, this discussion is consistent with Theorem 2 which requires that all BFB TQFT lines are self-dual and Abelian.^13^

We should distinguish between two separate cases of degenerate *S*:

1. $\mathcal {B}$ with a “slightly” degenerate *S* matrix (see the general discussion in [33]). In this case, we have a “super-MTC” with a single transparent line: a fermion, $\psi$, that generates $\mathcal {Z}_M(\mathcal {B})$
  2.20 $$\begin{aligned} \mathcal {Z}_M(\mathcal {B})\cong \langle \psi \rangle \cong \textrm{SVec} , \end{aligned}$$
  where $\textrm{SVec}$ is the category of finite-dimensional super vector spaces. A super-MTC is the algebraic realization of the line operators in a spin TQFT (i.e., a TQFT that depends on the spin structure of spacetime). In this setting, $\psi$ is a transparent fermion. We can for example realize $\textrm{SVec}$ via $SO(N)_1\cong \textrm{Spin}(N)_1/\mathbb {Z}_2$ CS theory, where we condense a fermionic $\mathbb {Z}_2$ line in $\textrm{Spin}(N)_1$ (e.g., see [34]).
2. $\mathcal {B}$ with any other degenerate *S* matrix. In this case, we should understand $\mathcal {B}$ as part of some non-topological QFT.

We will finish this section by describing case 1 above, and we will leave a discussion of case 2 to Sect. 2.2. To that end, in the first case, we have a super-MTC with the only non-trivial transparent line being the fermion, $\psi$. As follows from (2.19), this line does not contribute to $\nu _2(\ell _i)$, and we get

2.21 $$\begin{aligned} \nu _2(\ell _i)=d_{\ell _i}. \end{aligned}$$

In a unitary super-MTC, $d_{\ell _i}\ge 1$ for all $\ell _i \in \mathcal {B}$. However, for non-self-dual lines $\nu _2(\ell _i)=0$. Therefore, the above equality implies that in a super-MTC with real spins all lines must be self-dual,^14^ As a result, we learn that in a super-MTC, we again have (see also [30])

2.22 $$\begin{aligned} d_{\ell _i}=1 ,\ \ \ \forall \ell _i\in \mathcal {B}~. \end{aligned}$$

In other words, all BFB spin TQFTs are also Abelian. Moreover, all Abelian super-MTCs are split, which means any such super-MTC, $\mathcal {M}$, can be written as $\mathcal {M}\cong \widehat{\mathcal {M}}\boxtimes {\textrm{SVec}}$, where $\widehat{\mathcal {M}}$ is an MTC (see, for example, [35, 37]).

Therefore, using the classification in [21], we arrive at the following theorem:

### Theorem 3

The most general BFB spin TQFT corresponds to the following super-MTC (see also [30])

2.23 $$\begin{aligned} \mathcal {M}\cong (E_2)^{\boxtimes n}\boxtimes (F_2)^{\boxtimes m}\boxtimes \textrm{SVec} ,\ \ \ (X)^{\boxtimes p}:=\overbrace{X\boxtimes X\boxtimes \cdots \boxtimes X}^\mathrm{p\ times}~. \end{aligned}$$

In fact, using the equivalences $E_2\boxtimes \textrm{SVec}\cong F_2\boxtimes \textrm{SVec}$ and $E_2\boxtimes E_2\cong F_2\boxtimes F_2$, we can write any $\mathcal {M}$ as follows

2.24 $$\begin{aligned} \mathcal {M}\cong (E_2)^{\boxtimes n}\boxtimes \textrm{SVec}\cong (F_2)^{\boxtimes n}\boxtimes \textrm{SVec}~. \end{aligned}$$

At the level of CS theories, such an MTC can be realized by, for example, stacking *n* $\textrm{Spin}(N)_1$ CS theories ($N=0$ mod 16) with an $SO(M)_1$ CS theory or *n* $\textrm{Spin}(N')_1$ CS theories ($N'=8$ mod 16) with $SO(M')_1$ CS theory.

A few remarks are in order:

1. Theorems 2 and 3 hold even in a non-unitary (super-) MTC (see Appendix A for an argument).
2. The converses of Theorems 2 and 3 guarantee that any (spin) TQFT with non-invertible lines contains line operators with complex spins. Assuming the low-energy description of a general topological phase is a (spin) MTC, we have shown that if a topological phase contains anyons with non-invertible fusion rules, then it must contain anyons with complex spins.
3. In [14], two of the present authors asked whether time-reversal symmetry of a non-Abelian TQFT can act trivially on line operators. Theorems 2 and 3 answer this question in the negative.^15^

In the next section, we discuss how to realize the above BFB (spin) TQFTs via RG flows. Then, in Sect. 2.2, we discuss the case of more general BFB symmetries and give a classification.

### BFB (spin) TQFTs and UV completions

In general, given a (spin) TQFT, it is interesting to ask what kind of UV completion one can find. In our context, we have in mind a UV Poincaré-invariant and non-topological QFT, $\mathcal {Q}_{UV}$, that flows to the (spin) TQFT in the IR.^16^ Indeed, by better understanding such flows, one hopes to elucidate the structure of the space of QFTs. However, given a general class of abstract (spin) TQFTs, we do not expect it to be straightforward to find such a UV completion. On the other hand, we have seen in (2.16) and (2.23) that, by imposing Bose/Fermi statistics on (spin) TQFT lines, we get a remarkably simple class of theories.

Even in this context, a UV completion is wildly non-unique due to dualities of non-topological theories (e.g., see [39] for some relevant dualities that we will return to in Sect. 3.3). Examples of these dualities include IR dualities (i.e., where distinct UV theories flow to the same IR theory) and more trivial examples where distinct UV theories differ by some matter fields that can be made massive and integrated out.

Let us turn to some examples. Note from the discussion around (2.16) that we can realize the $E_2$ BFB MTC via $\textrm{Spin}(N)_1$ CS theory for $N=0$ mod 16. However, when we couple this theory to matter, it becomes non-topological and generally distinct for different values of *N*. For example, we can take $\textrm{Spin}(N)$ YM theory with CS level $k=1$ and couple $N_f$ real scalars, $\phi _a$ (with $a=1,\cdots ,N_f$), in the vector representation of the gauge group. This UV theory is clearly distinct for all $N=0$ mod 16. Then, giving a large mass to each of the matter fields, $\delta \mathcal {L}=-{m^2\over 2}\phi _a^2$, with $m\gg g^2$ (where *g* is the gauge coupling) results in dual $\textrm{Spin}(N)_1$ CS theories in the IR.

As a result, it is trivial to find UV completions for all BFB (spin) TQFTs. For example, we can engineer all MTCs in (2.16) by taking

2.25 $$\begin{aligned} \mathcal {Q}_{UV}:=(\textrm{Spin}(N)_1\ \textrm{with}\ N_f\ \phi \in \textbf{N})^{\boxtimes n}\boxtimes (\textrm{Spin}(N')_1\ \textrm{with}\ N_f\ \phi \in \mathbf{N'})^{\boxtimes m} , \end{aligned}$$

where $N=0$ mod 16 and $N'=8$ mod 16, and the scalars transform in the vector representation. Note that in these theories, a $\mathcal {B}_{UV}\cong \textrm{Rep}(\mathbb {Z}_2^{n+m})$ subcategory of lines is topological in the UV. This statement holds because the fundamental Wilson line can end on the matter fields and so the other would-be topological lines of each $\textrm{Spin}(N)_1$ and each $\textrm{Spin}(N')_1$ factor become non-topological (they braid non-trivially with at least one fundamental Wilson line while the fundamental Wilson lines braid trivially with themselves; e.g., see [40]).

**Fig. 3:**
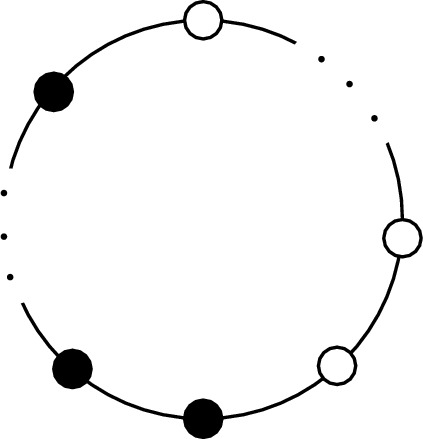
The circular quiver above describes a UV completion for each of the topological phases in (2.16) and (2.23) (note that we can couple in an *SO*(*M*) node with corresponding matter and appropriate level as well if desired). Black nodes denote $\textrm{Spin}(N)$ gauge groups (with $N=0\ \textrm{mod}\ 16$), and white nodes denote $\textrm{Spin}(N')$ gauge groups (with $N'=8\ \textrm{mod}\ 16$). For each gauge group, we turn on appropriate CS levels as described in the main text. Lines connecting nodes in the quiver correspond to appropriate matter fields (described in the main text) transforming as vectors under each of the corresponding two gauge groups. In this particular UV completion, we have made a choice to place the *n* black nodes in one grouping and the *m* white nodes in another (other arrangements are also acceptable; ours is universal in the sense that it exists for any *m* and *n*). Slightly away from zero gauge coupling, this theory has no decoupled sectors. Turning on large masses for the matter fields takes us to the topological phases described by (2.16) and (2.23)

Now, giving large mass to the scalars (compared to the squares of the individual gauge couplings) results in the theory described in (2.16) (the additional topological lines that emerge are accidental symmetries). If we want, we can also consider a $\mathcal {Q}_{UV}$ that does not have decoupled sectors in the UV. A simple way to do this is to consider gauge group $\textrm{Spin}(N)^n\times \textrm{Spin}(N')^m$ and construct a circular quiver (i.e., a circular graph with nodes corresponding to gauge groups and edges corresponding to matter) with $\phi$’s charged under successive gauge groups as bi-vector representations (i.e., for successive gauge nodes, $\phi \in \mathbf{(A,B)}$ for gauge group $\textrm{Spin}(A)\times \textrm{Spin}(B)$; see Fig. 3).^17^

To engineer UV completions for all super-MTCs in (2.16), we can instead consider

2.26 $$\begin{aligned} \mathcal {Q}_{UV}:=(\textrm{Spin}(N)_{1+N_f/2}\ \textrm{with}\ N_f\ \psi \in \textbf{N})^{\boxtimes n}\boxtimes (\textrm{Spin}(N')_{1+N_f/2}\ \textrm{with}\ N_f\ \psi \in \mathbf{N'})^{\boxtimes m} , \end{aligned}$$

where the $\psi _a$ are Majorana fermions, and the $N_f/2$ shifts in the UV levels arise from the massless fermion determinants. If we give large negative masses to the Majorana fermions, $\delta \mathcal {L}=-M\psi _a\bar{\psi }_a$, with $|M|\gg g^2$ and $M<0$, we obtain the theory in (2.23). As in the previous case, if we want a theory with a unique stress tensor, we can consider a circular quiver but replace the bosons with fermions (see Fig.  3).

### Non-(super-) modular BFB symmetries

In this section, we discuss the more general case of BFB symmetries in which the *S* matrix is non-degenerate and the Müger center satisfies

2.27 $$\begin{aligned} \mathcal {Z}_M(\mathcal {B})\not \cong \textrm{Vec} ,\ \textrm{SVec}~. \end{aligned}$$

In other words, we are interested in BFB symmetries in which we have transparent lines other than the trivial line and the transparent fermion. As we will discuss in more detail below, we should physically think of such $\mathcal {B}$ symmetries as corresponding to sectors of non-topological QFTs.^18^

Let us describe these $\mathcal {B}$ categories more carefully. As alluded to above, we know from Deligne’s theorem that $\mathcal {Z}_{M}\cong \textrm{Rep}(G_{z})$ for some non-trivial (super-) group, $G_{z}$. Here we use $G_{z}$ to refer to either a discrete group or a discrete super-group. In the case that there are no transparent fermions, $G_{z}$ is a discrete group, and we will write the group as $G_1$. Otherwise, we write $G_{\psi }$. Since the braiding of $\mathcal {Z}_M(B)$ is trivial, we always have a closed subcategory that includes all the bosonic lines [43]

2.28 $$\begin{aligned} \mathcal {Z}_{M}^\textrm{bos}(\mathcal {B}):=\textrm{Rep}(G)\le \textrm{Rep}(G_z)~. \end{aligned}$$

When there are no transparent fermions, $G\cong G_1$, and $\mathcal {Z}_M^\textrm{bos}(\mathcal {B})\cong \mathcal {Z}_M(\mathcal {B})$.

Therefore, given a general BFB category, $\mathcal {B}$, we are always free to condense a non-anomalous $\mathcal {Z}_{M}^\textrm{bos}(\mathcal {B})<\mathcal {B}$ (in the math literature this is referred to as de-equivariantization; e.g., see [10]). Indeed, this condensation is precisely the process that removes the correction terms in (2.19) (this condensation corresponds to a 0-gauging that is closely related to the 2-gauging described in footnote 13; in the 0-gauging, the relevant object is a restriction of *J* to elements in $\mathcal {Z}_M^\textrm{bos}(\mathcal {B})$). Clearly, this procedure produces an associated (super-) MTC

2.29 $$\begin{aligned} \mathcal {M}:=\mathcal {B}/\mathcal {Z}_{M}^\textrm{bos}(\mathcal {B})~. \end{aligned}$$

By the results in Sect. 2, $\mathcal {M}$ has the form given in (2.16) or (2.23) depending on whether there is a transparent fermion or not.

To better understand the landscape of allowed $\mathcal {B}$ categories, we should start with an $\mathcal {M}$ of the form in (2.16) or (2.23), construct an action of a discrete group, *G*, on this category and equivariantize (i.e., perform the inverse operation of condensation/de-equivariantization).^19^ This mathematical maneuver amounts to dropping twisted sectors and considering the splitting and fusing of the lines in $\mathcal {M}$ under the action of *G*. The reason we throw out twisted sectors is that we perform the inverse procedure of condensing a subcategory, $\mathcal {Z}_{M}^\textrm{bos}(\mathcal {B})$, that braids trivially with all the lines in $\mathcal {B}$ (and hence no lines are projected out in the de-equivariantization). However, there are sometimes obstructions to equivariantization (e.g., see [44]), which we will soon discuss from a physical perspective.

In any case, if we have an unobstructed *G*-equivariantization of an $\mathcal {M}$ of the form in (2.16) or (2.23), then the quantum dimensions in $\mathcal {B}$ are clearly integers (this statement is also consistent with the discussion in footnote 10). As a result, $\mathcal {B}$ is said to be “integral” [10].

It is also interesting to understand to what extent the theory of groups underlies our $\mathcal {B}$ categories. To that end, we note that the $\mathcal {M}$ (super-) MTCs in (2.16) and (2.23) are “group theoretical” since they are Morita equivalent to the representation category $\textrm{Rep}(\mathbb {Z}_2^p)$ for some $p>0$ (for super-MTCs we have $\textrm{Rep}(\mathbb {Z}_2^p)\rightarrow \textrm{Rep}(\mathbb {Z}_2^p)\boxtimes \textrm{SVec}$).^20^ These super-MTCs are therefore also “weakly” group theoretical in the sense of [10]. Since this latter property is preserved under equivariantization [10, Prop. 9.8.4], we have arrived at the following theorem:

#### Theorem 4

Given any BFB category, $\mathcal {B}$, there exists a unique discrete group, *G*, such that $\mathcal {B}$ can be constructed via a consistent *G*-equivariantization of a (super-) MTC, $\mathcal {M}$, of the form in (2.16) or (2.23). As a result, all BFB symmetries are integral and weakly group theoretical (in the sense of [10]). Moreover, since we can condense $\mathcal {Z}_M^\textrm{bos}(\mathcal {B})$ to get an invertible one-form symmetry, it also follows that BFB categories are non-intrinsically non-invertible (in the sense of [11, 12]).

In the next section, we would like to understand more precisely when a “consistent” *G*-equivariantization exists. To do so, we will mostly focus on the case $\mathcal {Z}_{M}^\textrm{bos}(\mathcal {B})\cong \mathcal {Z}_{M}(\mathcal {B})$ and invoke some physical reasoning. At the end of the next section, we will also discuss the case with transparent fermions.

### Coupling to QFTs, anomaly cancelation, and general BFB categories

In this section, we would like to understand the possible BFB symmetry categories more concretely. Except for some comments and an explicit example at the end of this section, we will mostly focus on the case

2.30 $$\begin{aligned} \mathcal {Z}_{M}^\textrm{bos}(\mathcal {B})\cong \mathcal {Z}_{M}(\mathcal {B})\not \cong \textrm{Vec}~. \end{aligned}$$

In other words, we will primarily study $\mathcal {B}$ symmetries that have no transparent fermions. We call such $\mathcal {B}$ symmetries “non-spin” BFB categories (generalizing the case of non-spin TQFTs). However, in what follows, we will often drop this modifier (instead, when there are cases with a transparent fermion, we will call such $\mathcal {B}$, “spin” BFB categories).

The main issue we wish to address is that the description of BFB categories via Theorem 4 is implicit and depends on the existence of a consistent *G*-equivariantization. Moreover, since *G*-equivariantization is closely related to gauging *G*, one may wonder about the role of *G* ’t Hooft anomalies. Therefore, we would like to first understand such categories more physically before deriving further theorems.

To that end, note that a $2+1$d QFT, $\mathcal {Q}$, should make sense on manifolds with $T^2$ spatial slices. On these manifolds, line operators in $\mathcal {Q}$ should be able to “detect” each other through their mutual statistics (e.g., see [46–49] and also [50] in the (spin) TQFT case). In particular, lines should not be completely “invisible.” When $\mathcal {Q}$ is a TQFT, this is the statement that the modular *S* matrix is invertible. When $\mathcal {Q}$ is a spin TQFT, *S* no longer needs to be invertible, but the mild degeneracy in this case reflects the existence of a transparent fermion that can be associated with the spin structure of the spacetime on which $\mathcal {Q}$ lives. This discussion explains our analysis of the $\mathcal {B}$ categories of Sect. 2.

In the case of more general $\mathcal {B}$ categories, the lines in $\mathcal {Z}_M^\textrm{bos}(\mathcal {B})$ are, by definition, “undetectable” to the degrees of freedom in $\mathcal {B}$. Therefore, in order for the lines in $\mathcal {Z}_M^\textrm{bos}(\mathcal {B})$ to not be invisible, they must act non-trivially on some non-topological lines in $\mathcal {Q}$. In other words, $\mathcal {B}<\mathcal {Q}$ should be a symmetry (sub) category for the non-topological QFT, $\mathcal {Q}$.

We can arrange for precisely such an embedding of $\mathcal {B}\hookrightarrow \mathcal {Q}$ as a symmetry by coupling $\mathcal {B}$ to a non-topological QFT, $\mathcal {T}$. In particular, recall that, by Theorem 4, any $\mathcal {B}$ is a *G*-equivariantization of an MTC, $\mathcal {M}$. This statement implied throwing out the twisted sectors of the *G* gauging of $\mathcal {M}$. We would like to not throw out such twisted sectors by hand and instead find a use for them.

**Fig. 4:**
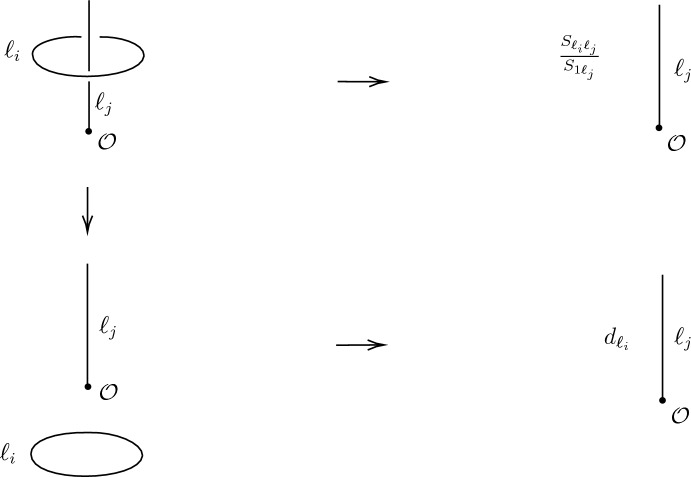
Shrinking a loop of topological line operator $\ell _{i}$ on $\ell _j$, we get $\frac{S_{\ell _{i} \ell _{j}}}{S_{1\ell _{j}}}$. Since $\ell _j$ is an endable line operator, we can move the $\ell _i$ loop down and then shrink it to get $d_{\ell _i}$. This shows that $\frac{S_{\ell _{i} \ell _{j}}}{S_{1\ell _{j}}}=d_{\ell _i}$. Therefore, if $\ell _i$ acts non-trivially on $\ell _j$, $\ell _i$ must be non-topological

To that end, suppose both $\mathcal {M}$ and $\mathcal {T}$ have an action of *G*. Moreover, assume that there are local operators in $\mathcal {T}$ transforming under all irreducible representations of *G*.^21^ Then, we can consider gauging the diagonal group, $G_\textrm{diag}\cong G\cong \textrm{diag}(G\times G)$, that couples $\mathcal {M}$ to $\mathcal {T}$ to produce the theory $\mathcal {Q}$. If the gauging is well-defined, then the twisted sector lines we produce from gauging $\mathcal {M}$ become non-topological lines in $\mathcal {Q}$, and only $\mathcal {B}$ lines remain as symmetries of $\mathcal {Q}$. The reason the twisted sector lines become non-topological is that these lines braid non-trivially with the “quantum” $\mathcal {Z}_M(\mathcal {B})\cong \textrm{Rep}(G)$ one-form symmetry that arises, and the $\textrm{Rep}(G)$ Wilson lines can end on local operators, $\mathcal {O}_i$, transforming under the corresponding irrep, $\pi _i\in \textrm{Rep}(G)$ [40] (note that the $\mathcal {B}$ lines braid trivially with the quantum symmetry; see Fig. 4). For example, if $\mathcal {Q}$ is a CFT, then we expect the twisted sector lines to become one-dimensional defect CFTs (DCFTs). Therefore, we have precisely accomplished, at the level of the topological lines, a *G*-equivariantization by endowing the twisted sector lines with non-trivial displacement operators.

But we should be careful to insist that the $G_\textrm{diag}$ gauging is well-defined. Indeed, this amounts to studying a theory in which the ’t Hooft anomaly, $\omega _4\in H^4(G_\textrm{diag},U(1))$ is vanishing in cohomology, and the so-called Postnikov class, $\beta \in H^3_{\rho }(G_\textrm{diag},\mathcal {A})$ is vanishing (here $\mathcal {A}$ consist of the invertible one-form symmetry lines).^22^

Let us consider $\omega _4\in H^4(G_\textrm{diag},U(1))$ first. We have that

2.31 $$\begin{aligned} \omega _4=\omega _4|_{\mathcal {M}}+\omega _4|_{\mathcal {T}} , \end{aligned}$$

where $\omega _4|_{\mathcal {M}}$ and $\omega _4|_{\mathcal {T}}$ are the contributions to the anomaly from $\mathcal {M}$ and $\mathcal {T}$ respectively. In general, it may happen that $\omega _4|_{\mathcal {M}}\ne [0]$ in cohomology. In this case, we will need the contribution from $\mathcal {T}$ to cancel it. We claim we can always arrange for such a cancelation to occur through an appropriate modification of $\mathcal {T}$. Indeed, since $H^4(G_\textrm{diag},U(1))$ is a finite Abelian group, $\omega _4|_{\mathcal {M}}$ and $\omega _4|_{\mathcal {T}}$ have order $n\ge 1$ and $m\ge 1$ respectively. We can now redefine

2.32 $$\begin{aligned} \mathcal {T}\rightarrow (\mathcal {T})^{\boxtimes m}~. \end{aligned}$$

In other words, we stack *m* copies of $\mathcal {T}$ together and call this our new theory, $\mathcal {T}$. We can extend the definition of $G_\textrm{diag}$ to act diagonally on each of the *m* copies of $\mathcal {T}$ as well so that $\omega _4|_{\mathcal {T}}=[0]$. To get rid of the anomaly from $\mathcal {M}$, we can now deform

2.33 $$\begin{aligned} \mathcal {T}\rightarrow \mathcal {T}\boxtimes (\mathcal {M})^{\boxtimes (n-1)} , \end{aligned}$$

and extend the action of $G_\textrm{diag}$ to the $(n-1)$ copies of $\mathcal {M}$ we have stacked onto $\mathcal {T}$. If we wish to render each copy of $\mathcal {M}$ non-topological, we can always couple it to sufficiently many free real scalars as we did in Sect. 2.1. In any case, we now have

2.34 $$\begin{aligned} \omega _4=\omega _4|_{\mathcal {M}}+\omega _4|_{\mathcal {T}}=[0]~. \end{aligned}$$

In particular, we see that the existence of an $H^4(G,U(1))$ anomaly in $\mathcal {M}$ does not matter. This statement is in fact consistent with the mathematical literature on equivariantization, which ignores such anomalies.

Next, let us discuss the Postnikov class, $\beta$. In this case, even after performing the modifications in (2.33) and (2.34), we cannot cancel $\beta \ne 0$. The reason is that this quantity is operator valued. In particular, $\beta$ is valued in $\mathcal {A}$, the invertible (part of the) one-form symmetry of $\mathcal {M}$. Adding additional copies of $\mathcal {M}$ and $\mathcal {T}$ therefore cannot cancel $\beta$. As a result, we should still insist on $\beta =0$. This statement is again consistent with the mathematical literature. According to the results in [52], the Postnikov class vanishes in gaugings of Abelian MTCs. As a result, we arrive at the following theorem:

#### Theorem 5

Assuming the results in [52] (see also [53] and references therein) on the vanishing of the Postnikov class in Abelian TQFTs (at least in theories of the type (2.16)), we find a one-to-one correspondence between non-spin BFBs and *G*-equivariantizations for any finite group, *G*, of an MTC of the form in (2.16).

One consequence of this theorem is that, in the non-(super-) modular case, $\mathcal {B}$ can have non-Abelian lines *and* non-trivial braiding. In other words, generic $\mathcal {B}$ BFB categories can, like the symmetric categories described by Deligne, have non-Abelian lines.

Moreover, Theorem 5 leads to an explicit description of the modular data of any non-spin BFB category in terms of the data of an MTC of the form in (2.16) and a *G*-action on it. Indeed, let $\mathcal {M}$ be an MTC of the form in (2.16). The action of a 0-form symmetry group *G* on an MTC is specified by the triple $(\rho ,\eta ,\alpha )$, where

2.35 $$\begin{aligned} \begin{aligned}&\rho : G \rightarrow \text {Aut}(\mathcal {M}) ,\\&\eta \in H^{2}(G,\text {Inv}(\mathcal {M})) ,\\&\alpha \in H^{3}(G,U(1))~. \end{aligned} \end{aligned}$$

Here $\rho$ is the symmetry action on $\mathcal {M}$, $\eta$ is the symmetry fractionalization class (specifying the local projective symmetry action), $\text {Inv}(\mathcal {M})$ is the subgroup of invertible 1-form symmetries in $\mathcal {M}$, and $\alpha$ is the SPT (or Dijkgraaf–Witten action) for *G*.^23^

From Theorem 5, we know that, upon gauging *G*, we get a non-spin BFB category, $\mathcal {B}$. We also have the freedom to stack a *G*-SPT while gauging the symmetry *G*. This maneuver changes the correlation functions of the twisted-sector line operators and leaves the data of the genuine topological line operators unchanged. Since the twisted-sector lines operators are non-topological in our case, stacking a *G*-SPT do not change the details of the topological line operators obtained after gauging *G*.

The line operators in $\mathcal {B}$ can be written as

2.36 $$\begin{aligned} ([\ell ],\pi _\ell ) , \end{aligned}$$

where $[\ell ]$ is an orbit of line operators in $\mathcal {B}$ under *G*-action with representative $\ell$, and $\pi _\ell$ are irreducible projective representations of the centralizer $C_\ell$ of $\ell$ satisfying

2.37 $$\begin{aligned} \pi _\ell (g) \pi _\ell (h) = ~\eta _\ell (g,h)~ \pi _\ell (gh) ~ \forall ~ g,h \in C_\ell ~. \end{aligned}$$

Note that the representations $\pi _{\ell }$ depend crucially on the choice of fractionalization class. In particular, if $\eta _{\ell }(g,h)$ is a non-trivial 2-cocycle on $C_{\ell }$, then all irreducible projective representations are higher-dimensional. The topological spin of these line operators is given by

2.38 $$\begin{aligned} \theta ((\ell ,\pi _\ell ))=\theta (\ell ) , \end{aligned}$$

where $\theta (\ell )$ is the spin of $\ell \in \mathcal {M}$. Also, the normalized *S* matrix of $\mathcal {B}$ is given by [51]

2.39 $$\begin{aligned} S_{([\ell _1],\pi _{\ell _1}),([\ell _2],\pi _{\ell _2})}= \frac{1}{|G|} \sum _{t\in G/G_{\ell _1}, s\in G/G_{\ell _2}} S_{{}^t{\ell _1},{}^s{\ell _2}} ~\text {dim}(\pi _{\ell _1}) \text {dim}(\pi _{\ell _2}) , \end{aligned}$$

where $G_{\ell }$ is the normal subgroup of *G* that stabilizes $\ell$. Finally, using the expression for fusion rules of equivariantizations of fusion categories in [55] (see also [51]), the fusion rules of $\mathcal {B}$ can be explicitly determined.

To conclude this section, let us comment on the case of spin BFB categories. From Theorem 4, we know that these can be obtained from equivariantization of a consistent *G*-action on a super-MTC of the form in (2.23). Even though the super-MTCs in (2.23) are all Abelian, unlike the case of Abelian MTCs, an arbitrary group *G* does not always act consistently on an Abelian super-MTC.

In particular, the generalization of the Postnikov class to *G*-actions on Abelian super-MTCs can be non-trivial. Several examples of this phenomenon are given in [44]. Let us construct an explicit example of *G*-action on a BFB spin TQFT with non-trivial Postnikov class.

To that end, consider the super-MTC, $\mathcal {M}:= D(\mathbb {Z}_{2})\boxtimes \{1,\psi \} = \{1,e,m,f\}\boxtimes \{1,\psi \}$, where $\psi$ is the transparent fermion. Let us focus on the $G_f \cong \mathbb {Z}_{2}\times \mathbb {Z}^{F}_{2}$ 0-form symmetry generated by $g_1$ and $(-1)^F$, where $g_1$ implements the transformation

2.40 $$\begin{aligned} 1\rightarrow 1 ,\ \ \ m\rightarrow m ,\ \ \ e \rightarrow f\psi ,\ \ \ f\rightarrow e\psi , \end{aligned}$$

and $\mathbb {Z}^{F}_{2}$ is the fermion parity generated by $(-1)^F$. We prescribe the following action of the symmetry on the fusion spaces

2.41 $$\begin{aligned} \rho _{g_1}[V^{a,b}] := U_{g_1}(a,b)V^{g_1(a),g_1(b)} ,\ \ \ U_{g_1}(a,b) := (-1)^{a_eb_e+a_eb_{\psi }} , \end{aligned}$$

where we denote the anyons of $\mathcal {M}$ as $a:= (a_e,a_m,a_\psi )$ with $a_i \in \{0,1\}$ (so that we have $a=e^{a_e}m^{a_m}\psi ^{a_{\psi }}$).^24^ The fractionalization class, $\nu (g,h) = (gh,0,0)$, has a fermionic Postnikov class given by  (here $d_{\rho }$ is the twisted differential of $C^{\star }_{\rho }(\mathbb {Z}_{2},\mathcal {M})$ complexes). In particular,  is non-trivial in $H^{3}(\mathbb {Z}_{2},\mathcal {M}/\{1,\psi \})$ (this fermionic Postnikov class corresponds to the obstruction discussed in [56, Section V.B.2] (see also [57]). This shows that the 1-form symmetry of $\mathcal {M}$ forms a fermionic version of a non-trivial 2-group with the $\mathbb {Z}_{2}\times \mathbb {Z}^{F}_{2}$ 0-form symmetry. Therefore, this 0-form symmetry cannot be gauged.

In the next section we will use the above results and study continuous deformations of non-topological $\mathcal {Q}$ with $\mathcal {B}$ symmetry.

## Continuous Deformations and RG Flows

In Sect. 2, we saw that all BFB categories can be obtained by stacking a highly restricted set of Abelian (super-) MTCs in (2.16) and (2.23) with a non-topological QFT and coupling them by gauging a diagonal finite 0-form *G* symmetry.^25^ Here we make use of this fact and discuss its implications for continuous deformations of non-topological QFTs. We will focus primarily on RG flows.

Let us suppose that we start with some UV QFT, $\mathcal {Q}_{UV}$, that has a one-form symmetry category, $\mathcal {B}_{UV}<\mathcal {Q}_{UV}$ (recall that, in our terminology, one-form symmetry can also be non-invertible). For now, we will assume that this category is a general premodular fusion category.^26^ We will return to the case where $\mathcal {B}_{UV}$ is a BFB category (or, slightly more generally, a BFB subcategory) soon. As we have seen in the previous section, via condensation of the transparent bosonic lines in the Müger center, $\mathcal {Z}_{M}^\textrm{bos}(\mathcal {B}_{UV})\le \mathcal {B}_{UV}$, we obtain a (super-) MTC, $\mathcal {M}_{UV}$

3.1 $$\begin{aligned} \mathcal {M}_{UV}:=\mathcal {B}_{UV}/\mathcal {Z}_M^\textrm{bos}(\mathcal {B}_{UV})~. \end{aligned}$$

The modular data of these two categories agree up to condensation of transparent lines (or, depending on which side one starts with, equivariantization).

Now, let us consider an RG flow which, for concreteness, starts from some UV-complete QFT (e.g., a CFT) and involves turning on vacuum expectation values for local operators (e.g., as is common in free bosonic theories or when going onto the moduli space of vacua in supersymmetric theories; this expectation value could also be the result of dynamics of the QFT) and/or turning on various local deformations. We expect such a flow to respect $\mathcal {B}_{UV}$, in the sense that the $\mathcal {B}_{UV}$ symmetry defects remain topological and remain as genuine lines (although some defects may become trivial).

More generally, our results apply to any flow that preserves $\mathcal {B}_{UV}$. It might seem surprising that we consider flows that allow some of the defects in $\mathcal {B}_{UV}$ to condense/trivialize (e.g., this can happen explicitly through turning on vacuum expectation values for defect endpoint operators). The point is that by “preserving $\mathcal {B}_{UV}$,” we mean that only condensation/trivialization of operators in $\mathcal {Z}_{M}(\mathcal {B}_{UV})$ can occur. In particular, none of the $\mathcal {B}_{UV}$ lines are (in the condensed matter language) confined or (in the high-energy theory language) rendered non-genuine as a result of the RG flow.^27^

**Fig. 5:**
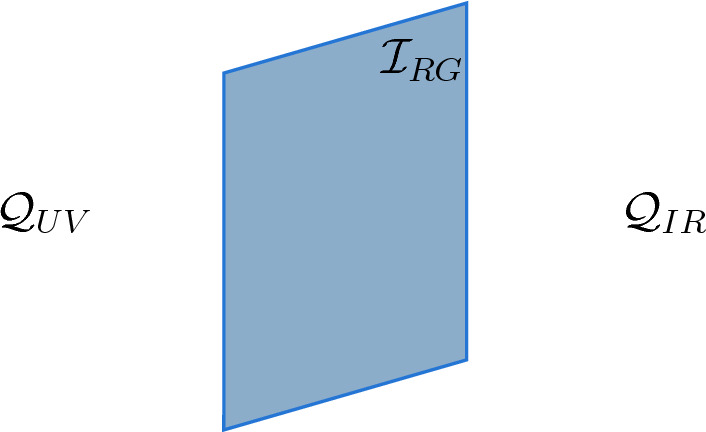
An RG interface, $\mathcal {I}_{RG}$, between the UV and IR QFTs. In principle, this interface encodes the mapping of all UV operators to IR operators

The result of this flow can be summarized as follows for the degrees of freedom of interest to us

3.2 $$\begin{aligned} \mathcal {B}_{UV}<\mathcal {Q}_{UV}\ \xrightarrow []{\text { RG }}\ F_{RG}(\mathcal {B}_{UV})<\mathcal {Q}_{IR} , \end{aligned}$$

where $F_{RG}$ is a functor, some of whose properties we will describe below, that implements the RG.^28^ In other words, $F_{RG}$ maps the UV QFT to the IR QFT

3.3 $$\begin{aligned} F_{RG}:\mathcal {Q}_{UV}\rightarrow \mathcal {Q}_{IR}~. \end{aligned}$$

The image, $F_{RG}(\mathcal {B}_{UV})$, of the UV one-form symmetry is in general only a proper subcategory of the IR one-form symmetry

3.4 $$\begin{aligned} F_{RG}(\mathcal {B}_{UV})\le \mathcal {B}_{IR}~. \end{aligned}$$

This statement holds because, although $F_{RG}(\mathcal {B}_{UV})$ is closed, we typically expect additional accidental/emergent symmetries in the IR (we have already seen examples of this common phenomenon in Sect. 2.1), and so $F_{RG}(\mathcal {B}_{UV})$ constitutes the IR symmetries that are “visible” in the UV.^29^ Of course, depending on the ’t Hooft anomalies, parts or all of $F_{RG}(\mathcal {B}_{UV})$ may be trivial at long distances.

Another useful perspective on $F_{RG}$ is to think of it as a non-topological interface, $\mathcal {I}_{RG}$, between $\mathcal {Q}_{UV}$ and $\mathcal {Q}_{IR}$ (see Fig. 5). When $\mathcal {Q}_{UV}$ is a CFT, we have a particularly simple picture^30^: we can generate $\mathcal {I}_{RG}$ by integrating a relevant deformation of $\mathcal {Q}_{UV}$ (and/or turning on a vacuum expectation value) on half the spacetime and flowing to the IR. Let $\mathcal {A}^{(1)}(\mathcal {B})$ be a collection of basis-independent data that determines the braided fusion category $\mathcal {B}$. In others words, it is a collection of basis-independent polynomials, *p*(*F*, *R*), in the *F* and *R* matrices (generalizing the modular data) that determines $\mathcal {B}$. $\mathcal {A}^{(1)}(\mathcal {B})$ forms a complete set of invariants of $\mathcal {B}$.^31^ ’t Hooft anomaly matching for the one-form symmetry is the statement that^32^

3.5 $$\begin{aligned} p_{UV}(F,R)= p_{IR}(F_{RG}(F),F_{RG}(R)) , \end{aligned}$$

for all $p_{UV}(F,R)\in \mathcal {A}^{(1)}(\mathcal {B}_{UV})$ where $p_{IR}(F_{RG}(F),F_{RG}(R))\in \mathcal {A}^{(1)}(\mathcal {B}_{IR})$. This discussion captures the idea that all the basis-independent data of the UV 1-form symmetry must be preserved in the IR via the functor $F_{RG}$.

Let us describe $F_{RG}$ in more detail and summarize some of its properties. In the following, we will denote simple line operators in $\mathcal {B}_{UV}$ by *x*, *y*, *z* and simple line operators in $\mathcal {B}_{IR}$ by *a*, *b*, *c*. Since we are considering RG flows which preserve the $\mathcal {B}_{UV}$ symmetry, $F_{RG}(x)$ must be a topological operator in $\mathcal {B}_{IR}$ for all $x\in \mathcal {B}_{UV}$. In general, the RG functor, $F_{RG}$, maps a UV simple line operator to a direct sum of simple line operators in the IR. Indeed, we have

3.6 $$\begin{aligned} F_{RG}(x)= \sum _{a\in \mathcal {B}_{IR}} N_{F_{RG}(x)}^a ~ a , \end{aligned}$$

where $N_{F_{RG}(x)}$ are non-negative integers. The image of $\mathcal {B}_{UV}$ under $F_{RG}$, denoted $F_{RG}(\mathcal {B}_{UV})$, is a subcategory of $\mathcal {B}_{IR}$. It is defined as the subcategory generated by all simple line operators, $a \in \mathcal {B}_{IR}$, such that $N_{F_{RG}(x)}^a\ne 0$ for some $x \in \mathcal {B}_{UV}$. What properties should $F_{RG}$ (and $\mathcal {I}_{RG}$) have in order to reproduce (3.5)? One natural class of functors that satisfy this condition are braided monoidal functors [73]. In Appendix B we describe these functors in more detail and show that they lead to the anomaly matching conditions (3.5). We also describe the implications for $\mathcal {I}_{RG}$. In particular, we prove the following theorem^33^:

### Theorem 6

Assuming that the RG functor, $F_{RG}$, is a braided monoidal functor, it satisfies

(i) $$N_{F_{RG}(x)F_{RG}(y)}^{F_{RG}(z)}=N_{xy}^z$$.
(ii) $$d_{F_{RG}(x)}=d_x$$.
(iii) If $N_{F_{RG}(x)}^a\ne 0$, then $\theta (a)=\theta (x)$.

Equations (i) and (ii) follow from the monoidal property of the functor $F_{RG}$. In particular, condition (ii) above implies that if there are topological line operators in $\mathcal {B}_{UV}$ with irrational quantum dimensions, then $\mathcal {B}_{IR}$ cannot be trivial. Therefore, this condition is an obstruction to having a trivially gapped IR phase.^34^ When all line operators in $\mathcal {B}_{UV}$ have integer quantum dimensions, then (i) and (ii) alone do not give an obstruction to a trivially gapped IR phase. However, combining (i), (ii), and (iii), we get the following important corollary:

### Corollary

The braiding of line operators in $\mathcal {B}_{UV}$ and $\mathcal {B}_{IR}$ are related as follows

3.7 $$\begin{aligned} S_{xy} = \sum _{a,b} N_{F_{RG}(x)}^a N_{F_{RG}(y)}^b~ S_{ab}~. \end{aligned}$$

In fact, the RHS of the above equation is equal to $S_{F_{RG}(x),F_{RG}(y)}$ [31, Lemma 2.4]. This statement agrees with the expectation that the braiding between the line operators *x* and *y* in the UV must be equal to the braiding between (generically non-simple) line operators $F_{RG}(x)$ and $F_{RG}(y)$ in the IR. Clearly, (3.7) is a special case of the anomaly matching condition (3.5). Indeed, the *S*-matrix does not fully capture the 1-form symmetry anomaly. For example, when transparent fermionic line operators are present, the symmetry is not gaugeable (without coupling to a spin structure) even though the S-matrix for these line operators is trivial. However, if the *S*-matrix is non-trivial, then the IR phase cannot be trivially gapped.

What more can we say in general? Let us study the image of $\mathcal {M}_{UV}$ under $F_{RG}$

3.8 $$\begin{aligned} F_{RG}(\mathcal {M}_{UV})\cong &   F_{RG}(\mathcal {B}_{UV}/\mathcal {Z}_M^\textrm{bos}(\mathcal {B}_{UV}))\cong F_{RG}(\mathcal {B}_{UV})/ F_{RG}(\mathcal {Z}^\textrm{bos}_M(\mathcal {B}_{UV}))\\\cong &   F_{RG}(\mathcal {B}_{UV})/ \mathcal {Z}_M^\textrm{bos}(F_{RG}(\mathcal {B}_{UV})) , \end{aligned}$$

where in the second equivalence we used the fact that RG flows commute with topological operations. In the third equality, we used (3.5).^35^ Note that $F_{RG}$ relates the *F*, *R* matrices of $\mathcal {M}_{UV}$ and $F_{RG}(\mathcal {M}_{UV})$ up to a basis transformation. Therefore,

3.9 $$\begin{aligned} \mathcal {A}^{(1)}(\mathcal {M}_{UV})\cong \mathcal {A}^{(1)}(F_{RG}(\mathcal {M}_{UV}))~. \end{aligned}$$

Since the categories in question are (super-) MTCs, the above equivalence implies that the modular *S* and *T* matrices match between the two categories. However, modular data does not determine a (super-) MTC [74], and is therefore insufficient to match anomalies in general.^36^

To proceed further, let us instead apply our discussion to the case we have focused on in Sect. 2, namely the situation in which $\mathcal {B}_{UV}$ is a BFB category.^37^ By the above discussion, $F_{RG}(\mathcal {B}_{UV})$ is also a BFB category. Therefore, both $\mathcal {M}_{UV}$ and $F_{RG}(\mathcal {M}_{UV})$ are of the form (2.16) or (2.23). Since these are Abelian (super-) MTCs, they are determined by their modular data and

3.10 $$\begin{aligned} \mathcal {M}_{UV}\cong F_{RG}(\mathcal {M}_{UV})\cong \mathcal {M}, \end{aligned}$$

where the equivalence should be understood up to the action of an invertible topological surface. It is then natural to think of $\mathcal {I}_{RG}$ and the associated functor, $F_{RG}$, as acting on the above MTC data as an invertible (possibly trivial) zero-form symmetry. In particular, $\mathcal {M}$ is an invariant of the RG flow. We therefore arrive at the following statement:

### Theorem 7

Consider an RG flow of the type discussed around (3.2) from a UV theory, $\mathcal {Q}_{UV}$, with a UV BFB one-form symmetry category, $\mathcal {B}_{UV}<\mathcal {Q}_{UV}$. Then, $\mathcal {M}:=\mathcal {B}_{UV}/\mathcal {Z}_M(\mathcal {B}_{UV})$ is an invariant of the RG flow. Moreover, if there are no emergent one-form symmetries, then $\mathcal {M}_{IR}:=\mathcal {B}_{IR}/\mathcal {Z}_M(\mathcal {B}_{IR})$ is isomorphic to $\mathcal {M}$.^38^,^39^

Let us study some consequences of this result. By definition we have (up to the action of an invertible topological surface)

3.11 $$\begin{aligned} \mathcal {B}_{UV}/\mathcal {Z}_M^\textrm{bos}(\mathcal {B}_{UV})\cong F_{RG}(\mathcal {B}_{UV})/\mathcal {Z}_M^\textrm{bos}(F_{RG}(\mathcal {B}_{UV}))~. \end{aligned}$$

Now, by Deligne’s theorem [18]

3.12 $$\begin{aligned} \mathcal {Z}_M^\textrm{bos}(\mathcal {B}_{UV})\cong \textrm{Rep}(G^{UV}) ,\ \ \ \mathcal {Z}_M^\textrm{bos}(F_{RG}(\mathcal {B}_{UV}))\cong F_{RG}(\textrm{Rep}(G^{UV}))\cong \textrm{Rep}(F_{RG}(G^{UV}))~. \end{aligned}$$

**Fig. 6:**
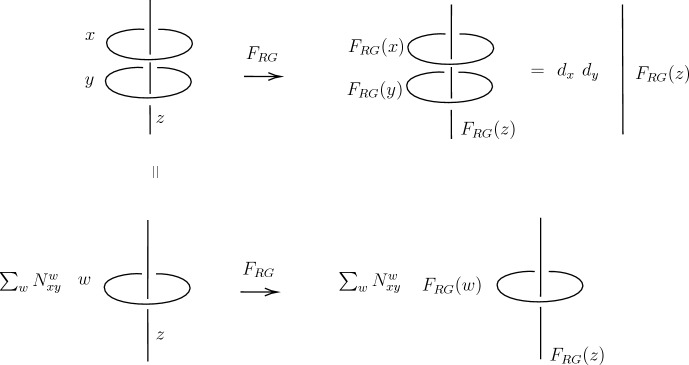
UV lines that are trivial in the IR are closed under fusion. Indeed, for the expressions in the two rows of the figure to agree, all $a\in F_{RG}(w)$ have to braid trivially with $F_{RG}(z)$ for all (topological and non-topological) line operators, *z*. As a result, all such *a* are trivial in the IR, and $w\in \mathcal {C}_\textrm{triv}$ for all $w\in x\times y$

Using the invertible map in (3.10), we can take the groups $G^{UV}$ and $F_{RG}(G^{UV})$ to act on $\mathcal {M}$. Moreover, along the RG flow, some lines forming a subcategory, $\mathcal {C}_\textrm{triv}\le \mathcal {Z}_M(\mathcal {B}_{UV})$, may become trivial in the IR (other lines in $\mathcal {B}_{UV}$ cannot trivialize due to their non-trivial braiding). More precisely, a line operator, $x \in \mathcal {B}_{UV}$, is said to trivialize in the IR if $F_{RG}(x)= d_{x}\cdot 1$. Note that the line operators in $\mathcal {C}_\textrm{triv}$ must be closed under fusion. To understand this statement, consider the line operator $x \times y$, where $x,y \in \mathcal {C}_\textrm{triv}$. Let us then study the braiding of $x \times y$ with some general (possibly non-topological) line operator, *z*. From Fig. 6, we find that the action of all simple lines in $x \times y$ on *z* must be trivial in the IR for any *z*. Therefore, $x \times y$ is trivial in the IR, and $\mathcal {C}_\textrm{triv}$ is a fusion subcategory, $\textrm{Rep}(G^{UV}/N)<\textrm{Rep}(G^{UV})$, for some normal subgroup, $N\lhd G^{UV}$.

Let us assume that any line operator $x\in \mathcal {B}_{UV}$ such that $F_{RG}(x)\ni 1$ satisfies $F_{RG}(x)=d_x\cdot 1$. Since the line operators in $\mathcal {C}_\textrm{triv}$ braid trivially with the genuine (topological or non-topological) line operators in the IR, the latter must have flux valued in the kernel of line operators in Rep$(G^{UV}/N)$. Demanding that the theory on $T^2\times \mathbb {R}$ is well-defined (we can also think of this requirement as a kind of generalization of the principle of remote detectability [46–49] for topological phases; see also [50]) suggests^40^

3.13 $$\begin{aligned} F_{RG}(\text {Rep}(G^{UV}))\cong \text {Rep}(N) , \end{aligned}$$

where $N=F_{RG}(G^{UV})$ is the normal subgroup of $G^{UV}$ described above, and

3.14 $$\begin{aligned} F_{RG}(\mathcal {B}_{UV})\cong \mathcal {B}_{UV}/\textrm{Rep}(G^{UV}/N)=\mathcal {B}_{UV}/\textrm{Rep}(G^{UV}/F_{RG}(G^{UV}))~. \end{aligned}$$

In particular, $F_{RG}(\mathcal {B}_{UV})$ and $\mathcal {B}_{UV}$ at most differ by anyon condensation.^41^ Equivalently, we can say that $\mathcal {I}_{RG}$ acts on the BFB category, $\mathcal {B}_{UV}$, as a (potentially trivial) surface implementing condensation from UV to IR combined with an invertible 0-form symmetry.

So far, we have assumed that some simple UV lines are completely trivialized in the flow to the IR (i.e., the situation in which $F_{RG}(x)=d_x\cdot 1$). More generally, simple UV lines can potentially be partially trivialized in the following sense

3.15 $$\begin{aligned} F_{RG}(x)=N_{F_{RG}(x)}^1\cdot 1+N_{F_{RG}(x)}^{a_0}\cdot a_0+\cdots ,\ \ \ N_{F_{RG}(x)}^1 ,\ N_{F_{RG}(x)}^{a_0}>0~. \end{aligned}$$

In writing (3.15), we have assumed that $a_0$ is a non-trivial simple IR line, and the ellipses contain any additional non-trivial IR contributions to $F_{RG}(x)$. We say that *x* is partially trivialized because $F_{RG}(x)$ contains a contribution from the trivial line. Such lines are part of a collection of lines we call $\mathcal {C}_\textrm{partial}$ (we can include $\mathcal {C}_\textrm{triv}$ as a potentially non-trivial subcategory). In general, $\mathcal {C}_\textrm{partial}$ is not closed under fusion. However, using the argument in footnote 40, we obtain

3.16 $$\begin{aligned} F_{RG}(\mathcal {Z}_M^\textrm{bos}(\mathcal {B}_{UV}))=F_{RG}(\text {Rep}(G^{UV}))=\text {Rep}(H) , \end{aligned}$$

where $H=F_{RG}(G^{UV})$ is now a general subgroup of $G^{UV}$. This argument shows that $\mathcal {Z}_M^\textrm{bos}(F_{RG}(\mathcal {B}_{UV}))=\text {Rep}(H)$. Moreover, $\mathcal {B}_{UV}$ and $F_{RG}(\mathcal {B}_{UV})$ are related by anyon condensation (the argument for this latter claim is similar to the one in footnote 41 but now $G^{UV}/H$ is a non-invertible symmetry; see also [79, Theorems 2.1, 2.2]). To summarize, we have:

### Theorem 8

Consider an RG flow of the type discussed around (3.2) from a UV theory, $\mathcal {Q}_{UV}$, with a UV BFB one-form symmetry category, $\mathcal {B}_{UV}$, to an IR theory, $\mathcal {Q}_{IR}$, with an IR BFB category, $\mathcal {B}_{IR}$. Then, $F_{RG}(\mathcal {B}_{UV})\le \mathcal {B}_{IR}$ differs from $\mathcal {B}_{UV}$ at most by anyon condensation.^42^

In Sect. 3.3, we will construct various examples that illustrate the above statement.^43^

Before continuing, let us make a comment on exactly marginal deformations in a continuous family of conformal field theories (e.g., the circle branch of the 2d compact boson or the gauge coupling fundamental domain in 4d $\mathcal {N}=4$ super Yang–Mills). Such excursions are close cousins of RG flows and, instead of an RG interface, we can consider an interface with different values of the exactly marginal coupling on each side (such interfaces are sometimes known as “Janus” interfaces [82]). In this case, we do not expect line operators to trivialize as we vary the exactly marginal parameter, and so it is natural to conjecture that if a CFT, $\mathcal {Q}$, with a BFB category, $\mathcal {B}<\mathcal {Q}$, is part of some conformal manifold, $M_{CFT}$, then $\mathcal {B}$ is an invariant of $M_{CFT}$.

In the next subsection we specialize to the case of gapped IR phases, where the long-distance physics is simpler, and we can therefore prove stronger statements.

### Gapped IR phases

Suppose the RG flow is such that the IR phase is gapped. Then, $\mathcal {B}_{IR}$ must be a (super-) MTC, $\mathcal {M}_{IR}$. There are various possibilities for the IR phase based on the fate of $\mathcal {Z}^\textrm{bos}_M(\mathcal {B}_{UV})$ under the RG flow. Let us consider two extreme cases:

1. Suppose the RG flow is such that all the lines in $\mathcal {Z}^\textrm{bos}_M(\mathcal {B}_{UV})$ get mapped to the trivial line in the IR. Then, the RG flow condenses $\mathcal {Z}_M^\textrm{bos}(\mathcal {B}_{UV})$ (we will see examples of this phenomenon in Sect. 3.3). As a result,
  3.17 $$\begin{aligned} \mathcal {B}_{IR}=\mathcal {M}_{IR}\cong \mathcal {M}\boxtimes \mathcal {M}' , \end{aligned}$$
  where $\mathcal {M}'$ may (or, depending on the details of the RG flow, may not) be a non-trivial (super-) MTC. Here we see that (a factor of) $\mathcal {B}_{IR}$ is the anomaly TQFT of $\mathcal {B}_{UV}$.
2. On the other hand, suppose all line operators in $\mathcal {B}_{UV}$ are mapped to simple IR lines. In particular, this statement implies that all the transparent lines in $\mathcal {Z}_M^\textrm{bos}(\mathcal {B}_{UV})$ are non-trivial in the IR. Therefore, the IR MTC, $\mathcal {M}_{IR}$, is a modular extension of $\mathcal {B}_{UV}$. Consider the image of $\mathcal {Z}_M^\textrm{bos}(\mathcal {B}_{UV})$, $F_{RG}(\mathcal {Z}_M^\textrm{bos}(\mathcal {B}_{UV}))<\mathcal {M}_{IR}$. Suppose the set of line operators in $\mathcal {M}_{IR}$ that braid trivially with all lines in $F_{RG}(\mathcal {Z}_M^\textrm{bos}(\mathcal {B}_{UV}))$ is precisely $F_{RG}(\mathcal {B}_{UV})$. In this case, the dimension of $\mathcal {M}_{IR}$ and $\mathcal {B}_{UV}$ are related as
  3.18 $$\begin{aligned} \text {dim}(\mathcal {M}_{IR})= \text {dim}(\mathcal {B}_{UV})\cdot \text {dim}(\mathcal {Z}_M^\textrm{bos}(\mathcal {B}_{UV}))~. \end{aligned}$$
  To understand this relation, condense the bosons $F_{RG}(\mathcal {Z}_M^\textrm{bos}(\mathcal {B}_{UV}))<\mathcal {M}_{IR}$. Only the line operators in $\mathcal {M}_{IR}$ which braid trivially with $F_{RG}(\mathcal {Z}_M^\textrm{bos}(\mathcal {B}_{UV}))$ survive this condensation. After condensation, we therefore obtain the MTC, $\mathcal {M}$. Therefore, $\mathcal {M}_{IR}$ can be obtained from gauging a 0-form symmetry, $G^{UV}$, of $\mathcal {M}$, and we have [51]
  3.19 $$\begin{aligned} \text {dim}(\mathcal {M}_{IR})=\text {dim}(\mathcal {M})\cdot |G^{UV}|^2~. \end{aligned}$$
  On the other hand, if we gauge the $G^{UV}$ symmetry of $\mathcal {M}$ without including twisted sectors, we get the category $\mathcal {B}_{UV}$, and so
  3.20 $$\begin{aligned} \text {dim}(\mathcal {B}_{UV})=\text {dim}(\mathcal {M})\cdot |G^{UV}|~. \end{aligned}$$
  Combined with the equation above, we get
  3.21 $$\begin{aligned} \text {dim}(\mathcal {M}_{IR})=\text {dim}(\mathcal {B}_{UV})\cdot |G^{UV}|= \text {dim}(\mathcal {B}_{UV})\cdot \text {dim}(\mathcal {Z}_M^\textrm{bos}(\mathcal {B}_{UV})) ~. \end{aligned}$$
  In the second equality we used the fact that $F(\mathcal {Z}_M^\textrm{bos}(\mathcal {B}_{UV}))\cong \text {Rep}(G^{UV})$ and that $\text {dim}(\mathcal {Z}_M^\textrm{bos}(\mathcal {B}_{UV}))=\text {dim}(\text {Rep}(G^{UV}))=|G^{UV}|$. $\mathcal {M}_{IR}$ is called a minimal modular extension of $\mathcal {B}_{UV}$ [31] (see also [83]). All lines in $\mathcal {M}_{IR}$ which are not in $F(\mathcal {B}_{UV})$ have a non-trivial mixed anomaly with lines in $F(\mathcal {Z}_M^\textrm{bos}(\mathcal {B}_{UV}))$. However, not all braided fusion categories, $\mathcal {B}_{UV}$, admit a minimal modular extension. For simplicity, we will focus on the case without transparent fermions.^44^ Following [35, 44], the obstruction to the existence of a minimal modular extension can be understood as follows. Condense $\mathcal {Z}_M^\textrm{bos}(\mathcal {B}_{UV})=\mathcal {Z}_M(\mathcal {B}_{UV})$ to get the MTC, $\mathcal {M}$, with a 0-form symmetry, $G^{UV}$. The action of $G^{UV}$ on the category $\mathcal {M}$ is guaranteed to have trivial Postnikov class. Now, $\mathcal {M}$ can be extended to a $G^{UV}$-crossed braided category, $\mathcal {M}_{G^{UV}}$, if and only if the anomaly of $G^{UV}$, $\omega \in H^4(G^{UV},U(1))$, is trivial. If the anomaly is trivial, then $\mathcal {M}_{G^{UV}}$ is a consistent $G^{UV}$-crossed braided category with a $G^{UV}$ action. This $G^{UV}$ action can be gauged to get an MTC, and the resulting MTC is the required minimal modular extension. However, if $G^{UV}$ is anomalous, then $\mathcal {B}_{UV}$ does not admit a minimal modular extension. Therefore, if $\mathcal {B}_{UV}$ is such that the $G^{UV}$-symmetry of $\mathcal {M}$ is anomalous, we see that the IR MTC, $\mathcal {M}_{IR}$, necessarily hosts line operators having trivial mixed-anomaly with $F_{RG}(\mathcal {Z}_M^\textrm{bos}(\mathcal {B}_{UV}))$ beyond those in $F_{RG}(\mathcal {B}_{UV})$. As a result [31, 44],
  3.22 $$\begin{aligned} \text {dim}(\mathcal {M}_{IR})= &   \textrm{dim}(C_{\mathcal {M}_{IR}}(F_{RG}(Z_M^\textrm{bos}(\mathcal {B}_{UV}))))\cdot \text {dim}(F_{RG}(\mathcal {Z}_M^\textrm{bos}(\mathcal {B}_{UV})))\\> &   \text {dim}(\mathcal {B}_{UV})\cdot \text {dim}(\mathcal {Z}_M^\textrm{bos}(\mathcal {B}_{UV})) , \end{aligned}$$
  where $C_{\mathcal {M}_{IR}}(F_{RG}(Z_M^\textrm{bos}(\mathcal {B}_{UV})))$ is the centralizer of $F_{RG}(\mathcal {Z}_M^\textrm{bos}(\mathcal {B}_{UV}))<\mathcal {M}_{IR}$ (i.e., the subcategory of lines in $\mathcal {M}_{IR}$ that braid trivially with $F_{RG}(\mathcal {Z}_M^\textrm{bos}(\mathcal {B}_{UV}))$).^45^ In fact, we can elaborate on the role of the additional lines, $\ell _i\in C_{\mathcal {M}_{IR}}(F_{RG}(\mathcal {B}_{UV}))$, that are not in $F_{RG}(\mathcal {B}_{UV})$. It is easy to see that their images under anyon condensation are precisely the lines needed to cancel the $G^{UV}$ anomaly. Indeed, condensing $\mathcal {Z}^\textrm{bos}_{M}(F_{RG}(\mathcal {B}_{UV}))$ yields
  3.23 $$\begin{aligned} \mathcal {M}_{IR}/\mathcal {Z}_{M}^\textrm{bos}(F_{RG}(\mathcal {B}_{UV}))=\mathcal {M}\boxtimes \mathcal {M}' , \end{aligned}$$
  where $\mathcal {M}'$ must be an MTC (coming from the images of the $\ell _i$ under condensation) with a $G^{UV}_{\psi }$ anomaly, $\omega '\in H^4(G^{UV},U(1))$, that cancels the anomaly, $\omega \in H^4(G^{UV}, U(1))$, arising from $\mathcal {M}$
  3.24 $$\begin{aligned} \omega +\omega '=[0]\in H^4(G^{UV},U(1)) , \end{aligned}$$
  so that we can gauge a diagonal $G^{UV}$ acting on $\mathcal {M}$ and $\mathcal {M}'$ to produce $\mathcal {M}_{IR}$.

As we saw in Sect. 3, the most general scenario is that the RG flow maps $\mathcal {Z}_M(\mathcal {B}_{UV})\cong \textrm{Rep}(G^{UV})$ to $F_{RG}(\mathcal {Z}_M^\textrm{bos}(\mathcal {B}_{UV}))\cong \textrm{Rep}(F_{RG}(G^{UV}))$, where $F_{RG}(G^{UV})<G^{UV}$ is a subgroup (the first case above corresponds to $F_{RG}(G^{UV})=\mathbb {Z}_1$, and the second case corresponds to $F_{RG}(G^{UV})\cong G^{UV}$).

We can repeat the analysis of the second case above in this more general setting. To that end, suppose that the set of line operators in $\mathcal {M}_{IR}$ that braid trivially with all lines in $F_{RG}(\mathcal {Z}_M^\textrm{bos}(\mathcal {B}_{UV}))$ is precisely $F_{RG}(\mathcal {B}_{UV})$. In this case, the dimension of $\mathcal {M}_{IR}$ and $\mathcal {B}_{UV}$ are again related as

3.25 $$\begin{aligned} \text {dim}(\mathcal {M}_{IR})= \text {dim}(F_{RG}(\mathcal {B}_{UV}))\cdot \text {dim}(F_{RG}(\mathcal {Z}_M^\textrm{bos}(\mathcal {B}_{UV})))~. \end{aligned}$$

We again have that $\mathcal {M}_{IR}$ is a minimal modular extension of $F_{RG}(\mathcal {B}_{UV})$ (although with respect to a subgroup, $F_{RG}(G^{UV})<G^{UV}$; if $F_{RG}(G^{UV})\cong \mathbb {Z}_1$, then $\mathcal {M}_{IR}\cong \mathcal {M}\cong F_{RG}(\mathcal {B}_{UV})$).

However, as in the second case above, $F_{RG}(\mathcal {B}_{UV})$ may not admit a minimal modular extension, and so

3.26 $$\begin{aligned} \text {dim}(\mathcal {M}_{IR})= &   \textrm{dim}(C_{\mathcal {M}_{IR}}(F_{RG}(Z_M(\mathcal {B}_{UV}))))\cdot \text {dim}(F_{RG}(\mathcal {Z}_M^\textrm{bos}(\mathcal {B}_{UV})))\\> &   \text {dim}(F_{RG}(\mathcal {B}_{UV}))\cdot \text {dim}(F_{RG}(\mathcal {Z}_M(\mathcal {B}_{UV})))~. \end{aligned}$$

Once more, $\mathcal {M}_{IR}$ has line operators with trivial mixed-anomaly with $F_{RG}(\mathcal {Z}_M^\textrm{bos}(\mathcal {B}_{UV}))$ beyond those in $F_{RG}(\mathcal {B}_{UV})$.

The role of the additional lines, $\ell _i\in C_{\mathcal {M}_{IR}}(F_{RG}(\mathcal {B}_{UV}))$, that are not in $F_{RG}(\mathcal {B}_{UV})$ generalizes their role in the previous case. Indeed, condensing $\mathcal {Z}_{M}^\textrm{bos}(F_{RG}(\mathcal {B}_{UV}))$ again yields

3.27 $$\begin{aligned} \mathcal {M}_{IR}/\mathcal {Z}_{M}^\textrm{bos}(F_{RG}(\mathcal {B}_{UV}))=\mathcal {M}\boxtimes \mathcal {M}' , \end{aligned}$$

where $\mathcal {M}'$ must be an MTC (coming from the image of the $\ell _i$) with an $F_{RG}(G^{UV})$ anomaly, $\omega '\in H^4(F_{RG}(G^{UV}),U(1))$, cancelling the anomaly, $\omega \in H^4(F_{RG}(G^{UV}), U(1))$, arising from $\mathcal {M}$

3.28 $$\begin{aligned} \omega +\omega '=[0]\in H^4(F_{RG}(G^{UV}),U(1)) , \end{aligned}$$

so that we can gauge a diagonal $F_{RG}(G^{UV})$ acting on $\mathcal {M}$ and $\mathcal {M}'$ to produce $\mathcal {M}_{IR}$.

We can summarize the above discussion via the following theorem:

#### Theorem 9

Consider a QFT, $\mathcal {Q}_{UV}$, with a one-form symmetry category, $\mathcal {B}_{UV}$. Let us imagine an RG flow of the type described above (3.2) emanating from $\mathcal {Q}_{UV}$ and ending in a gapped IR phase described by a (super-) MTC, $\mathcal {M}_{IR}$. Then, we have

3.29 $$\begin{aligned} \mathcal {M}_{IR}/\mathcal {Z}_{M}^\textrm{bos}(F_{RG}(\mathcal {B}_{UV}))=\mathcal {M}\boxtimes \mathcal {M}' , \end{aligned}$$

where $\mathcal {M}:=\mathcal {B}_{UV}/\mathcal {Z}_{M}^\textrm{bos}(\mathcal {B}_{UV})$. As a result, $\mathcal {M}_{IR}$ is obtained by gauging a diagonal $F_{RG}(G^{UV})$ zero-form symmetry such that the corresponding ’t Hooft anomalies of $\mathcal {M}$ and $\mathcal {M}'$ cancel.

Consider the special case when $\mathcal {B}_{UV}$ is a BFB category. In this case, $F_{RG}(\mathcal {B}_{UV})$ contains line operators with real spins. From Theorems 2 and 3, we know that a (super-) MTC with non-invertible line operators must contain line operators with complex spin. Therefore, when $F_{RG}(\mathcal {B}_{UV})$ contains at least one non-invertible line operator, we find that the MTC $\mathcal {M}_{IR}$, which is a modular extension of $F_{RG}(\mathcal {B}_{UV})$, contains emergent line operators with complex spin.

### Lines of $\mathcal {M}$ as ’t Hooft spectators

In this section, we would like to further comment on the role that $\mathcal {M}:=\mathcal {B}_{UV}/\mathcal {Z}^\textrm{bos}_M(\mathcal {B}_{UV})$ plays in the above RG flow discussion. One aspect of $\mathcal {M}$ that we have explained in great detail in Sect. 3 is that it encodes the one-form anomalies of $\mathcal {B}_{UV}$ and $F_{RG}(\mathcal {B}_{UV})$.

A more subtle role that $\mathcal {M}$ plays is hinted at in Sect.  3.1: the lines of $\mathcal {M}$ are zero-form anomaly “spectators” reminiscent of the weakly coupled spectator fields appearing in the original argument for ’t Hooft anomaly matching [16].

To understand this analogy, first recall that ’t Hooft’s spectators, $\mathcal {S}$, are free fields charged under a global symmetry Lie group, *G*, that a QFT, $\mathcal {T}_{UV}$, whose dynamics is being studied, is also charged under. Now, suppose that $\mathcal {T}_{UV}$ has an anomaly $\mathcal {A}^{(0)}(\mathcal {T}_{UV})\ne 0$ and that the spectators have cancelling anomaly, $\mathcal {A}^{(0)}(\mathcal {S})=-\mathcal {A}^{(0)}(\mathcal {T}_{UV})$. Then, the diagonal *G* acting on the combined $\mathcal {S}$ and $\mathcal {T}_{UV}$ degrees of freedom can be gauged. Moreover, we can consider gauging this symmetry in a parametrically weak way (i.e., taking the coupling at a given energy to be arbitrarily small). After initiating the RG flow, the resulting IR theory, $\mathcal {T}_{IR}$, coming from $\mathcal {T}_{UV}$ is generally very different (i.e., with emergent degrees of freedom), but $\mathcal {S}$ will consist of the same weakly coupled UV fields in the IR because the gauging is parametrically weak (we can then consider “ungauging” the symmetry to produce decoupled $\mathcal {S}$ and $\mathcal {T}_{IR}$ sectors without affecting the dynamics). Finally, since $\mathcal {A}^{(0)}(\mathcal {S})$ does not change, we learn that $\mathcal {A}^{(0)}(\mathcal {T}_{UV})=\mathcal {A}^{(0)}(\mathcal {T}_{IR})$.

In our case, we can produce $\mathcal {B}_{UV}<\mathcal {Q}_{UV}$ by gauging a diagonal $G^{UV}$ symmetry of $\mathcal {M}$ and $\mathcal {T}_{UV}$. As in ’t Hooft’s discussion, we will generally have non-vanishing but cancelling anomalies for $G^{UV}$ in $\mathcal {M}$ and $\mathcal {T}_{UV}$. While we cannot weakly gauge a discrete symmetry as ’t Hooft did for continuous symmetries, the fact that $\mathcal {M}$ has non-vanishing one-form anomaly means that it is always present along the RG flow. More precisely, the logic around (3.10) shows it is an invariant of an appropriate condensation/zero-form “ungauging.” Moreover, while the UV and IR zero-form symmetry groups $G^{UV}$ and $F_{RG}(G^{UV})\le G^{UV}$ can be different, the IR anomaly $F_{RG}(\omega )\in H^4(F_{RG}(G^{UV}),U(1))$ for $\mathcal {M}$ is non-zero only if the UV anomaly $\omega \in H^4(G^{UV},U(1))$ is non-vanishing.

### Examples

Let us now construct examples that illustrate the discussion in Sects. 3, 3.1, and 3.2. Our examples are all UV-complete Poincaré-invariant theories coupled to topological degrees of freedom. We then study RG flows characterized by turning on vacuum expectation values and/or local deformations.

We choose examples of $\mathcal {Q}_{UV}$ belonging to at least one of the following two classes of non-topological QFTs that contain topological lines (note that there is not always an invariant distinction between these classes; for example, dualities can relate them):

1. Theories built from gauging a diagonal discrete symmetry, $G_D$, of a TQFT of the form (2.16) or (2.23) and a CFT (the TQFT may also be trivial, in which case we are coupling a $G_D$ discrete gauge theory with Dijkgraaf–Witten twist, $\omega _3\in H^3(G_D,U(1))$, to the CFT). Depending on which representations of $G_D$ are present in the CFT, we will have different spectra of topological and non-topological lines. For example, if we have a local CFT operator transforming in representation $R\in \textrm{Rep}(G_D)$, then the Wilson line, $W_R$, of charge *R* can end on this operator. As a result, topological line operators that braid non-trivially with $W_R$ become non-topological [40] (since the bulk theory is a CFT, the topological lines that braid non-trivially with $W_R$ become one-dimensional defect CFTs). If $G_D$ is non-Abelian (or if $\omega _3$ is suitably chosen), we can have non-Abelian topological lines.
2. Theories corresponding to *G* Yang–Mills (YM) theories with CS terms at level $\vec {k}$ (here $\vec {k}$ is a vector of levels that depends on *G*) coupled to charged massless matter (we will often refer to these theories as “$G_{\vec {k}}$ QCD theories”). Such QFTs can be understood as relevant deformations of free gauge and matter fields. For generic matter representations, these QFTs have no topological lines. However, if the matter representations are neutral under the center of *G*, *Z*(*G*), then there is an Abelian one-form symmetry group implemented by lines with fusion rules isomorphic to *Z*(*G*) [9]. We can sometimes engineer non-Abelian topological lines in such theories as follows. Suppose we have a $G_{\vec {k}}$ QCD theory with Abelian topological lines arising from *Z*(*G*). Let us now gauge an outer automorphism, $G_D$, of *G* to produce a gauge group corresponding to an extension, $\tilde{G}$. If the matter fields live in representations that lift to $\tilde{G}$ and if the Abelian topological lines form non-trivial orbits under the outer automorphism, we find non-Abelian topological lines [84].^46^ Since this procedure can be thought of as gauging a discrete symmetry of $G_{\vec {k}}$ QCD (which, depending on the theory, can even flow to a CFT in the IR^47^), it can also be thought of as an example of the previous class of theories. Note that we can also, as in the first class of theories, consider gauging a diagonal $G_D$ symmetry of $G_{\vec {k}}$ QCD and one of the TQFTs of the form (2.16) or (2.23).

Starting from $\mathcal {Q}_{UV}$ in (at least) one of the above classes of QFTs, we can then imagine turning on two different types of locality preserving deformations (or a combination thereof; note that, under duality, these kinds of deformations are often exchanged):

1. Relevant deformations like mass terms (or more general ones). At energy scales small compared to the mass, we typically find various emergent symmetries. If the massive charged matter is fermionic, then the Chern-Simons levels and Dijkgraaf–Witten twists shift at one-loop with signs determined by the signs of the fermionic mass terms and magnitude determined by the amount of matter (measured by the Dynkin index, *T*(*R*), corresponding to the representation, $R\in \textrm{Rep}(G)$ that the matter is charged under).
2. We can sometimes turn on a vacuum expectation value for certain bosonic operators. Such deformations always exist in free bosonic CFTs and also in many supersymmetric theories because their potentials have (quantum protected) flat directions parameterized by gauge-invariant local operators. If $\mathcal {O}$ is a defect endpoint operator for some topological line, $\ell$, we can often find an *n* such that the normal-ordered product $:\mathcal {O}^n:$ is a genuine (gauge-invariant) local operator.^48^ Then, going to a vacuum in which $\langle :\mathcal {O}^n:\rangle \ne 0$ results in a condensation of $\ell$. This is one way in which the RG flow can trivialize topological lines (see also the discussion above (3.2)).

Let us consider some of the simplest examples of the first type discussed above. To that end, we take a real scalar

3.31 $$\begin{aligned} S(\phi )={1\over 2}\int d^3x\ \partial ^{\mu }\phi \partial _{\mu }\phi , \end{aligned}$$

and gauge the $\mathbb {Z}_2\cong \langle g\rangle$ symmetry that acts on $\phi$ as $g(\phi )=-\phi$. We can think of this gauging as coupling a $\mathbb {Z}_2$ SPT characterized by $\omega _3\in H^3(\mathbb {Z}_2, U(1))\cong \mathbb {Z}_2$ to $\phi$3.32In (3.32), “$(\mathbb {Z}_2)$” denotes the gauge group, and the links indicate that the degrees of freedom connected to $(\mathbb {Z}_2)$ have a $\mathbb {Z}_2$ global symmetry that has been gauged. The resulting theory has $\mathcal {B}_{UV}\cong \textrm{Rep}(\mathbb {Z}_2)$ generated by a Wilson line, $W_{(1,0)}$, that can end on $\phi$. Therefore, the remaining lines, $W_{(0,1)}$ and $W_{(1,1)}$, become non-topological DCFTs, because they braid non-trivially with $W_{(1,0)}$.

Let us now turn on a mass, $\delta \mathcal {L}=-{1\over 2}m^2\phi ^2$, and flow to the deep IR. We find (up to various countertetms) a (twisted) $\mathbb {Z}_2$ discrete gauge theory, $D(\mathbb {Z}_2)_{\omega _3}$

3.33 $$\begin{aligned} \mathcal {Q}_{IR}:={1\over 4\pi }\int d^3x {\vec {a}}^T Kd\vec {a} ,\ \ \ K=\begin{pmatrix} 0 &  2 \\ 2 &  2\omega _3 \\ \end{pmatrix} ,\ \ \ \omega _3=0, 1~. \end{aligned}$$

In this case, $F_{RG}(\mathcal {B}_{UV})\cong \mathcal {B}_{UV}\cong \textrm{Rep}(\mathbb {Z}_2)$, and $\mathcal {B}_{IR}\cong \mathcal {Q}_{IR}\cong \mathcal {M}_{IR}$ (anomalies match rather trivially between the UV one-form symmetry and the non-emergent part of the IR one-form symmetry). Therefore, it is clear that (3.29) holds with $\mathcal {M}_{IR}/F_{RG}(\mathcal {B}_{UV})$ a trivial TQFT (i.e., both $\mathcal {M}$ and $\mathcal {M}'$ are trivial).^49^

Since $\mathcal {Q}_{UV}$ has a ray of vacua, $\mathcal {V}\cong \mathbb {R}_{\ge 0}$, parameterized by $\langle :\phi ^2:\rangle$, we can consider going to points on this space with $\langle :\phi ^2:\rangle \ne 0$ (i.e., the second class of deformations described above). In this case, we condense $\mathcal {B}_{UV}$ (i.e., $F_{RG}(\mathcal {B}_{UV})\cong \textrm{SPT}(\mathbb {Z}_2)_{\omega _3}$) and find that $\mathcal {B}_{IR}\cong \textrm{SPT}(\mathbb {Z}_2)_{\omega _3}$ is trivial as a theory of lines. This discussion is again consistent with (3.29).

Let us now consider examples where $\mathcal {B}_{UV}$ has some non-trivial braiding. To that end, let us couple an untwisted $\mathbb {Z}_2$ discrete gauge theory, $D(\mathbb {Z}_2)$, to a free real scalar3.34where the action of $\mathbb {Z}_2\cong \langle g\rangle$ is EM duality on $D(\mathbb {Z}_2)$ and sign-flip on $\phi$

3.35 $$\begin{aligned} g(\ell _{(e,m)})=\ell _{(m,e)} ,\ \ \ g(\phi )=-\phi ~. \end{aligned}$$

The $\omega _3$ subscript in (3.34) describes the $\mathbb {Z}_2$ SPT we turn on in the process of gauging the $\mathbb {Z}_2$ symmetry in (3.35).

In $\mathcal {Q}_{UV}$, we have the following topological lines

3.36 $$\begin{aligned} \mathcal {B}_{UV}\cong \left\{ \ell _{(0,0),\pm } , \ \ell _{(1,1),\pm } ,\ \ell _{[(1,0)]}\right\} ,\ \ \ \mathcal {Z}_M(\mathcal {B}_{UV})\cong \left\{ \ell _{(0,0),\pm }\right\} \cong \textrm{Rep}(\mathbb {Z}_2) , \end{aligned}$$

where $\ell _{(0,0),-}$ is transparent to the other topological lines (i.e., it generates the Müger center of $\mathcal {B}_{UV}$) and can end on $\phi$ (as described in Sect. 2, this fact guarantees that the twisted sector lines become non-topological), $\ell _{(1,1),\pm }$ are fermionic Abelian lines that come from the dyon under $\mathbb {Z}_2$ gauging, and $\ell _{[(1,0)]}$ is the quantum dimension-two line that comes from the $\mathbb {Z}_2$ orbit, $\ell _{(1,0)}\oplus \ell _{(0,1)}$. Clearly $\mathcal {B}_{UV}$ is a $\mathbb {Z}_2\times \mathbb {Z}_2$ Tambara–Yamagami (TY) category with modular data

3.37 $$\begin{aligned} S=\begin{pmatrix} 1 &  1 &  1&  1 &  2\\ 1 &  1 &  1&  1 &  2\\ 1 &  1 &  1&  1 &  -2\\ 1 &  1 &  1&  1 &  -2\\ 2 &  2 &  -2&  -2 & 0\\ \end{pmatrix} ,\ \ \ \theta (\ell _{(0,0),\pm })=\theta (\ell _{[(1,0)]})=1 ,\ \theta (\ell _{(1,1),\pm })=-1~.\qquad \end{aligned}$$

Now, let us consider turning on a mass, $\delta \mathcal {L}=-{1\over 2}m^2\phi ^2$, and flowing to the IR. We find

3.38 $$\begin{aligned} \mathcal {B}_{IR}\cong {\mathcal {Q}}_{IR}\cong \mathcal {M}_{IR}\cong {\textrm{Ising}}^{(\nu )}\boxtimes \overline{{\textrm{Ising}}^{(\nu )}} , \end{aligned}$$

where $\nu =1,3$ label different theories related to the $\mathbb {Z}_2$ SPT we stack with in (3.34) (we can choose $\nu =2\omega _3+1$). In particular, $\nu =1$ corresponds to what is typically referred to as the Ising TQFT (i.e., we have the topological spin $\theta (\sigma )=\exp (\pi i/8)$ for the non-invertible line), and $\nu =3$ corresponds to $\textrm{Ising}^{(3)}\cong SU(2)_2$. More generally, $\nu =1,3,5,7$, but we have the identifications $\nu =1\sim \nu =7$ and $\nu =3\sim \nu =5$ in the IR product theory (3.38).

Clearly, we also have that $F_{RG}(\mathcal {B}_{UV})\cong \mathcal {B}_{UV}$, and so this example is consistent with the general non-Abelian anomaly matching discussion in the main text. In fact, we can re-write the UV theory as3.39where the coupling to $\textrm{Ising}\boxtimes \overline{\textrm{Ising}}$ is via the $(\epsilon ,\overline{\epsilon })$ line.^50^

We can also consider turning on a VEV by moving out onto the $\mathcal {V}\cong \mathbb {R}_{\ge 0}$ moduli space of the free scalar by turning on $\langle :\phi ^2:\rangle \ne 0$. This maneuver gives us $D(\mathbb {Z}_2)\boxtimes \textrm{SPT}(\mathbb {Z}_2)_{\omega _3}\boxtimes S(\phi )$, and we find

3.40 $$\begin{aligned} \mathcal {B}_{IR}=F_{RG}(\mathcal {B}_{UV})=D(\mathbb {Z}_2)\cong \mathcal {B}_{UV}/\mathcal {Z}_{M}(\mathcal {B}_{UV})~. \end{aligned}$$

This example is again consistent with our general discussion in the previous sections.

Next let us consider $\textrm{Pin}^+(N)$ Yang–Mills (YM) theory with bare CS level $k_0$ coupled to $N_f$ adjoint Majorana fermions (e.g., see [39, 85] for recent discussions of this theory). Let us define the shifted CS coupling $k:=k_0-(N-2)N_f/2$. Turning on $\delta \mathcal {L}=-M\Psi _a\bar{\Psi }_a$ and taking $|M|\gg g^2$ large (where *g* is the YM coupling), we have

3.41 $$\begin{aligned} \mathcal {B}_{IR}\cong \mathcal {Q}_{IR}\cong \mathcal {M}_{IR}\cong {\left\{ \begin{array}{ll} \mathrm{{Pin}}^+(N)_{k_0} \boxtimes \textrm{SVec}&  M>0 , \\ \textrm{Pin}^+(N)_{k_0-(N-2)N_f} \boxtimes \textrm{SVec}&  M<0 , \end{array}\right. } \end{aligned}$$

where the IR TQFT arises through the one-loop shift in the CS coupling.

For simplicity, let us focus in more detail on the case of $N=4$ and $k=k_0-N_f$. We can understand $\textrm{Pin}^+(4)_k$ as a gauging of the spin-exchange symmetry of $\textrm{Spin}(4)_k\cong SU(2)_k\times SU(2)_k$ (i.e., the symmetry that sends lines $(j_1,j_2)\leftrightarrow (j_2,j_1)$ where $0\le j_i\le k/2$), and (3.41) becomes

3.42 $$\begin{aligned} \mathcal {B}_{IR}\cong \mathcal {Q}_{IR}\cong \mathcal {M}_{IR}\cong {\left\{ \begin{array}{ll} \mathrm{{Pin}}^+(4)_{k_0} \boxtimes \textrm{SVec}&  M>0 , \\ \textrm{Pin}^+(4)_{k_0-2N_f} \boxtimes \textrm{SVec}&  M<0~. \end{array}\right. } \end{aligned}$$

If we start with a $\textrm{Spin}(4)_{k_0}$ theory coupled to $N_f$ adjoint Majorana fermions, the lines corresponding to the $\mathbb {Z}_2\times \mathbb {Z}_2$ center (i.e., $W_{(0,0)}$, $W_{(k_0/2,0)}$, $W_{(0,k_0/2)}$, and $W_{(k_0/2,k_0/2)}$) are topological even in the presence of the adjoint fermions. Then, so are the lines obtained by gauging the spin exchange symmetry to produce the $\textrm{Pin}^+(4)_{k_0}$ gauge theory coupled to $N_f$ adjoint fermions.^51^ Indeed, we have a $\mathbb {Z}_2\times \mathbb {Z}_2$ Tambara–Yamagami (TY) category

3.43 $$\begin{aligned} W_{[(k_0/2,0)]}\times W_{[(k_0/2,0)]}=W_{(0,0),+}+W_{(0,0),-}+W_{(k_0/2,k_0/2),+}+W_{(k_0/2,k_0/2),-} , \end{aligned}$$

where $W_{(0,0),-}$ is the quantum Abelian line that arises from gauging the exchange symmetry of the $\textrm{Spin}(4)_{k_0}$ theory, $W_{(k_0/2,k_0/2),\pm }$ are the images of the invariant $W_{(k_0/2,k_0/2)}$ $\textrm{Spin}(4)$ line under gauging, and $W_{[(k_0/2,0)]}$ is the non-Abelian line that arises from the $W_{(k_0/2,0)}\oplus W_{(0,k_0/2)}$ orbit under the exchange symmetry (see also the related discussion in [87]). Note that

3.44 $$\begin{aligned} \mathcal {B}_{UV}\cong \left\{ W_{(0,0),\pm } ,\ W_{(k_0/2,k_0/2),\pm }~,\ W_{[(k_0/2,0)]}\right\} \boxtimes \textrm{SVec} ~, \end{aligned}$$

and we find the following modular data for the TY lines in (3.42) (this subcategory is a BFB category for $k_0=2n$)

3.45 $$\begin{aligned} S= &   {\left\{ \begin{array}{ll}\begin{pmatrix} 1 & \quad 1 & \quad 1& \quad 1 & \quad 2\\ 1 & \quad 1 & \quad 1& \quad 1 & \quad 2\\ 1 & \quad 1 & \quad 1& \quad 1 & \quad -2\\ 1 & \quad 1 & \quad 1& \quad 1 & \quad -2\\ 2 & \quad 2 & \quad -2& \quad -2 & \quad 0\\ \end{pmatrix}&  k_0\ \textrm{odd}\\ \\ \begin{pmatrix} 1 & \quad 1 & \quad 1& \quad 1 & \quad 2\\ 1 & \quad 1 & \quad 1& \quad 1 & \quad 2\\ 1 & \quad 1 & \quad 1& \quad 1 & \quad 2\\ 1 & \quad 1 & \quad 1& \quad 1 & \quad 2\\ 2 & \quad 2 & \quad 2& \quad 2 & \quad 4\\ \end{pmatrix}&  k_0\ \textrm{even}\\ \end{array}\right. }~,\nonumber \\ \theta \left( W_{[(k_0/2,0)]}\right)= &   \exp (\pi i k_0/2)~,\ \ \ \theta \left( W_{(0,0),\pm }\right) =1~,\nonumber \\  &   \theta \left( W_{(k_0/2,k_0/2),\pm }\right) =\exp (\pi i k_0)~.\ \ \ \ \ \ \ \end{aligned}$$

Crucially, we see that the one-form symmetry anomalies match since the two IR phases are related by a shift in the IR CS level by an even number (i.e., $\delta k_{IR}=-2N_f$). Note that if $N_f$ is odd, then there is a mismatch between $\theta (\ell )$ in the two IR phases with the different signs for *M*. However, this mismatch is cancelled by including the $\textrm{SVec}$ factor (i.e., we map $\ell \leftrightarrow \ell \times \psi$ via a domain wall between the two IR phases, where $\psi \in \textrm{SVec}$ is the transparent fermion).

As our final example, consider the following case that illustrates the role of $\mathcal {M}\cong \mathcal {M}_{UV}$ as a generalization of the ’t Hooft spectators described in Sect. 3.23.46Here our notation for $\mathcal {Q}_{UV}$ means that we gauge the diagonal $\mathbb {Z}_2\times \mathbb {Z}_2\cong \langle g_1,g_2\rangle$ 0-form symmetry that acts on a complex free scalar as

3.47 $$\begin{aligned} \phi :={1\over \sqrt{2}}(\phi _1+i\phi _2)~,\ \ \ g_1(\phi )={1\over \sqrt{2}}(-\phi _1+i\phi _2)~,\ \ \ g_2(\phi )={1\over \sqrt{2}}(\phi _1-i\phi _2)~. \end{aligned}$$

This symmetry does not permute any of the lines in the $D(\mathbb {Z}_2)$ factors (note that one $D(\mathbb {Z}_2)$ factor is in $\mathcal {T}_{UV}$, so we can think of $\mathcal {B}_{UV}$ as a closed subcategory of the full UV one-form symmetry, $\mathcal {C}_{UV}:=D(\mathbb {Z}_2)^{\boxtimes 2}\boxtimes \textrm{Rep}(\mathbb {Z}_2\times \mathbb {Z}_2)$) but has an $H^4(\mathbb {Z}_2\times \mathbb {Z}_2, U(1))\cong \mathbb {Z}_2\times \mathbb {Z}_2$ anomaly in each factor. Indeed, following the discussion in [88], we can choose an anomaly $\omega ^{(i)}\in H^4(\mathbb {Z}_2\times \mathbb {Z}_2,U(1))$ in the *i*th $D(\mathbb {Z}_2)$ sector specified by the 4d action

3.48 $$\begin{aligned} S_{4d} = -\frac{2}{(2\pi )^2}\int A^{(i)}_1\,A^{(i)}_2\,(dA^{(i)}_1+dA^{(i)}_2)~, \end{aligned}$$

where the $A_{1,2}^{(i)}$ are background gauge fields for the $G^{(i)}\cong \mathbb {Z}_2\times \mathbb {Z}_2$ symmetry acting on the *i*th $D(\mathbb {Z}_2)$ sector. By considering a diagonal symmetry (i.e., identifying $A_a^{(1)}=A_a^{(2)}=A_a$) we obtain a vanishing anomaly (the contribution from the scalar sector is vanishing), and we can gauge the diagonal $\mathbb {Z}_2\times \mathbb {Z}_2$ symmetry described in (3.47).

Now, consider deforming the complex scalar action by $\delta \mathcal {L}=-m^2|\phi |^2$ and flowing to the IR. Via the RG flow, this maneuver renders the one-hundred-and-ninety-two UV DCFTs that we get from the twisted sector gauging of the two $D(\mathbb {Z}_2)$ theories topological in the IR, and we have an IR MTC with

3.49 $$\begin{aligned} |\mathcal {M}_{IR}|=256~,\ \mathcal {M}_{IR}\supset D(\mathbb {Z}_2)^{\boxtimes 2}\boxtimes \textrm{Rep}(\mathbb {Z}_2\times \mathbb {Z}_2)~, \ \mathcal {C}_{\mathcal {M}_{IR}}(\textrm{Rep}(\mathbb {Z}_2\times \mathbb {Z}_2))=D(\mathbb {Z}_2)^{\boxtimes 2}~. \end{aligned}$$

Here $\mathcal {M}_{IR}$ is a non-minimal modular extension of $\mathcal {B}_{UV}$.^52^ Clearly, the additional lines that braid trivially with $\textrm{Rep}(\mathbb {Z}_2\times \mathbb {Z}_2)$ come from the cancelling of the $H^4(\mathbb {Z}_2\times \mathbb {Z}_2,U(1))$ anomaly via $D(\mathbb {Z}_2)\subset \mathcal {T}_{UV}$.

Now let us consider the example from the previous bullet but, instead of adding a mass term, let us move onto the moduli space, $\mathcal {V}:=\mathbb {R}_+^2$, parameterized by $\langle :\phi _a^2:\rangle$. We find that $\mathcal {M}_{UV}=D(\mathbb {Z}_2)\rightarrow D(\mathbb {Z}_2)^{\boxtimes 2}=\mathcal {M}_{IR}$. In particular, $\mathcal {M}_{IR}$ still has accidental symmetries due to the anomaly. Note that the IR consists of a gapped phase stacked with a gapless one.

## Discussion

In this paper, we have classified BFB symmetry categories and shown that they are closely related to groups. In particular, we proved that any BFB category, $\mathcal {B}$, is weakly group theoretical and, if non-invertible, it is in fact non-intrinsically non-invertible. We have also showed that QFTs with BFB symmetries posses invariants associated with (locality preserving) continuous deformations (e.g., RG flows initiated by turning on expectation values and/or adding relevant local operators to the action). In particular, Theorems 6 (i), (ii), 7 and 9 follow from natural assumptions on the RG flow preserving the fusion and braiding between line operators. For RG flows in which some UV line operators are completely trivialized in the IR, we argued that the UV line operators and its image in the IR under the RG flow are related by anyon condensation. Moreover, using the technical assumption that $F_{RG}$ is a braided monoidal functor, we derived an explicit expression for the anomaly matching condition for 1-form symmetries (Theorem 6 (iii) and its Corollary). Under this assumption, we also derived Theorem 8 which showed that even in the general case when some UV line operators are only partially trivialized, the UV line operators are related to their IR images by anyon condensation.

Our work leaves many open questions. For example:

- As alluded to in the previous paragraph, we associated a (super-) MTC, $\mathcal {M}$, with each locality-preserving deformation of a QFT with BFB symmetry. Topological modular forms (TMFs) are also deformation invariants of large classes of QFTs (e.g., see [89–91]). Both our MTC and TMFs make use of modularity. Is there a deeper relation?
- Can we use the $\mathcal {M}$ invariants of the previous bullets to prove or conjecture new RG monotonicity theorems in $2+1$d?
- From [92, 93], we know that we can associate a quantum code with each Abelian MTC. Therefore, we realize a goal described in [92, 93] of associating quantum codes with deformation classes of QFTs. It would be interesting to further explore the implications of the resulting code map.
- We obtained our BFB categories by coupling to a non-topological QFT. It would be interesting to relate these ideas to the lattice constructions in [94, 95] and mixed state topological order.
- Our categories play important roles in dualities involving CS theories for groups, *G*, with $\mathfrak {so}(n)$ Lie algebras. Can our results be used to extend these dualities? Can we leverage known classification results involving metaplectic categories to further constrain these dualities?^53^
- In a similar spirit, BFB categories play an important role in the dualities and dynamics of 3d $\mathcal {N}=1$ theories in [96]. Can our results shed further light on these QFTs?

We hope to return to some of these questions soon.

## Figures and Tables

**Fig. 7:**
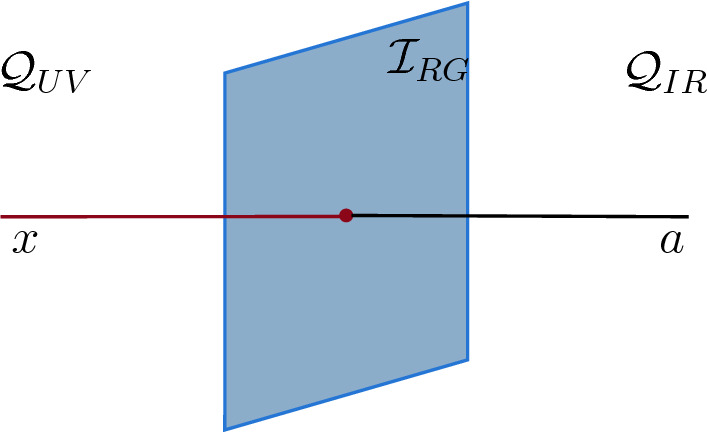
Junction of topological line operators on $\mathcal {I}$

**Fig. 8:**
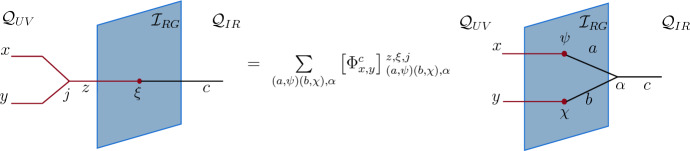
Map between fusion spaces under $\mathcal {I}_{RG}$

**Fig. 9:**
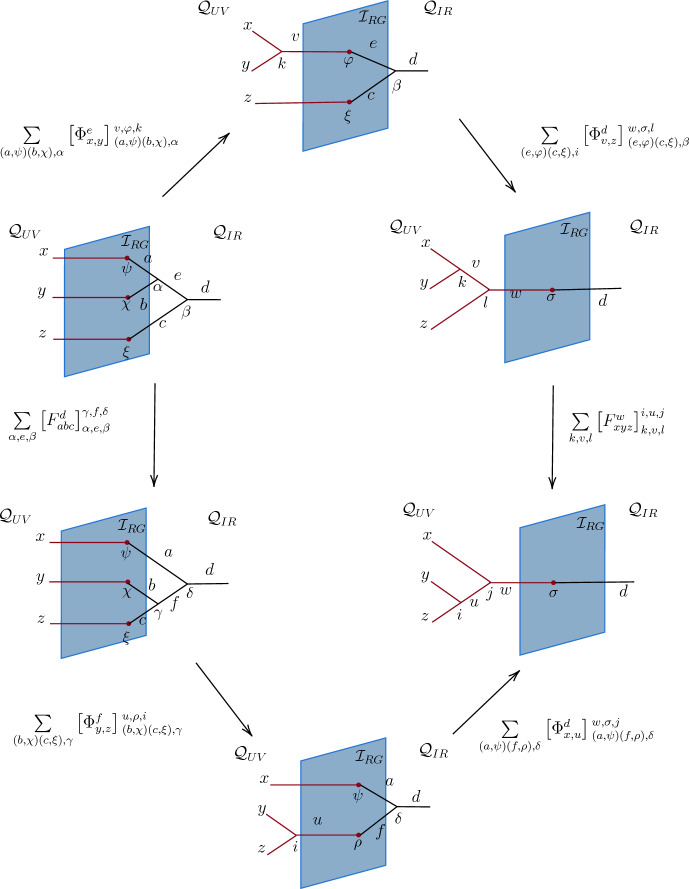
Compatibility of $\mathcal {I}_{RG}$ with the fusion of line operators

**Fig. 10:**
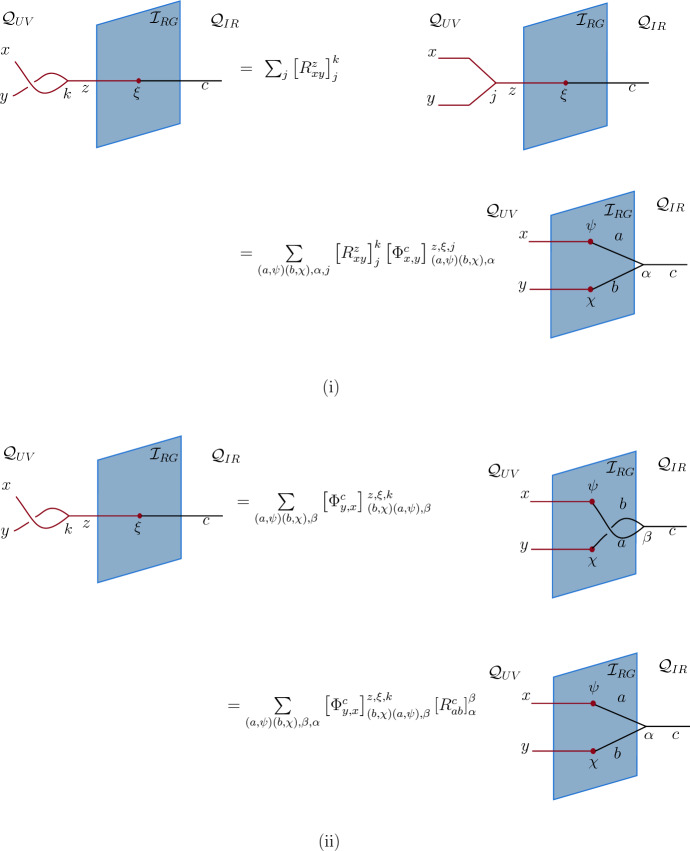
Compatibility of $\mathcal {I}_{RG}$ with the braiding of line operators

## Data Availability

No new data were generated or analyzed in this study.

## Non-unitary BFB (Super-) MTCs

The expression for the FS indicator given in (2.19) is also valid in a non-unitary super-MTC. Therefore, we can repeat the arguments leading to Theorems 2 and 3 to show that in a non-unitary BFB (super-) MTC,^54^

A.1 $$\begin{aligned} d_{\ell _i}=\nu _2(\ell _i)=\pm 1~ \forall ~ \ell _i \in \mathcal {B}~. \end{aligned}$$

In order to show that all line operators are invertible, we have to show that FPdim$(\ell _i)=1$ for all $\ell _i\in \mathcal {B}$.^55^ We will now show that this is indeed the case.

To that end, consider the modular tensor category, $\mathcal {Z}(\mathcal {B})$, obtained from taking the Drinfeld centre of the super-MTC, $\mathcal {B}$. We have the following relation

A.2 $$\begin{aligned} \text {FPdim}(\mathcal {Z}(\mathcal {B}))=\text {FPdim}(\mathcal {B})^2 ~, ~ D_{\mathcal {Z}(\mathcal {B})}=D^2_{\mathcal {B}}~, \end{aligned}$$

where FPdim$(\mathcal {B}):=\sum _{\ell _i\in \mathcal {B}} \text {FPdim}(\ell _i)^2$, and $D_{\mathcal {B}}:=\sqrt{\sum _{\ell _i\in \mathcal {B}} d_{\ell _i}^2}$. Recall that both quantum dimensions and Frobenius–Perron dimensions of line operators in $\mathcal {Z}(\mathcal {B})$ are one-dimensional characters of the $\mathcal {Z}(\mathcal {B})$ fusion ring. Since $\mathcal {Z}(\mathcal {B})$ is modular, there exists some line operator, $\ell _j \in \mathcal {Z}(\mathcal {B})$, such that

A.3 $$\begin{aligned} \text {FPdim}(\ell _i)=\frac{S_{\ell _i\ell _j}}{S_{1\ell _j}}~. \end{aligned}$$

Then, we get

A.4 $$\begin{aligned} \text {FPdim}(\mathcal {Z}(\mathcal {B}))=\sum _{\ell _i} \text {FPdim}(\ell _i)^2 = \sum _{\ell _i} \frac{S^2_{\ell _i\ell _j}}{S^2_{1\ell _j}} = \frac{D^2_{\mathcal {Z}(\mathcal {B})}}{S_{1\ell _j}^2}= \frac{D^2_{\mathcal {Z}(\mathcal {B})}}{d_{\ell _j}^2}~. \end{aligned}$$

Using (A.2), we get

A.5 $$\begin{aligned} \text {FPdim}(\mathcal {B})^2 =\frac{D_{\mathcal {B}}^4}{d_{\ell _j}^2}~. \end{aligned}$$

By assumption, the (super-) MTC, $\mathcal {B}$, has line operators with real spins. Using (A.1) and [27, Theorem 5.5], we find that the order of the *T*-matrix of $\mathcal {Z}(\mathcal {B})$ is 2. Since the quantum dimensions of $\mathcal {Z}(\mathcal {B})$ must live in the same number field as the topological spins [98, Proposition 4], this implies that the quantum dimensions of line operators in $\mathcal {Z}(\mathcal {B})$ are integers. Therefore, the $d_{\ell _j}^2$ in (A.5) is a positive integer. We get

A.6 $$\begin{aligned} \text {FPdim}(\mathcal {B})^2\le D_{\mathcal {B}}^4 \implies \text {FPdim}(\mathcal {B})\le D^2_{\mathcal {B}}~, \end{aligned}$$

where we have used the fact that FPdim$(\mathcal {B})$ is a positive number by definition, and $D_{\mathcal {B}}$ is positive in our case since $d_{\ell _i}=\pm 1 ~ \forall ~\ell _i\in \mathcal {B}$. Now, we use Proposition 8.21 in [99], which states that $D^2_{\mathcal {B}}\le \text {FPdim}(\mathcal {B})$ for any fusion category $\mathcal {B}$. Applying this to (A.5), we find that

A.7 $$\begin{aligned} \text {FPdim}(\mathcal {B})=D^2_{\mathcal {B}}~. \end{aligned}$$

Therefore, we find that any (super-) MTC with real spins is pseudo-unitary. By [99, Proposition 8.23], in any pseudo-unitary category there is a unique spherical structure such that quantum dimensions are positive and agree with the Frobenius-Perron dimensions. In our case, the allowed quantum dimensions are valued in $\pm 1$. As a result, there exists a spherical structure such that $d_{\ell _i}=1=\text {FPdim}(\ell _i) ~ \forall ~ \ell _i \in \mathcal {B}$, and all line operators in $\mathcal {B}$ are invertible.

## Details of the RG Interface and Braided Monoidal Functor

In this appendix, we will explicitly describe the RG functor

B.1 $$\begin{aligned} F_{RG}: \mathcal {B}_{UV} \rightarrow \mathcal {B}_{IR}~, \end{aligned}$$

and the assumptions that make it a braided monoidal functor. We will also give a proof of the anomaly matching condition.

To that end, let *x*, *y*, *z* be the simple line operators in $\mathcal {B}_{UV}$, and let *a*, *b*, *c* be the simple line operators in $\mathcal {B}_{IR}$. As discussed in Sect. 3, we have

B.2 $$\begin{aligned} F_{RG}(x)= \sum _a N_{F_{RG}(x)}^a ~ a~, \end{aligned}$$

for some non-negative integers, $N_{F_{RG}(x)}^a$. It is useful to think of the RG flow as an interface, $\mathcal {I}_{RG}$, between $\mathcal {Q}_{UV}$ and $\mathcal {Q}_{IR}$. In particular, $\mathcal {I}_{RG}$ tells us how the full spectrum of local and extended operators in $\mathcal {Q}_{UV}$ get mapped to $\mathcal {Q}_{IR}$. A subset of the data specifying $\mathcal {I}_{RG}$ is the map, $F_{RG}$, that tells us how the topological lines operators of the UV QFT are mapped to the IR QFT. If $N_{F_{RG}(x)}^a\ne 0$, then the line operators *x* and *a* can form a non-trivial junction on $\mathcal {I}_{RG}$ (see Fig. 7).

If $N_{xy}^z$ is non-zero, then the line operators *x*, *y*, *z* can form a tri-valent junction, which can host point operators belonging to a fusion space, $V_{xy}^z$, of dimension $N_{xy}^z$. As we move this fusion space across $\mathcal {I}_{RG}$, we get a fusion space, $V_{F_{RG}(x)F_{RG}(y)}^{F_{RG}(z)}$, in the IR QFT. Let us make the following assumptions about the RG interface:

- Since the fusion of two topological line operators does not depend on the distance between them, the RG flow must preserve fusion rules of line operators in $\mathcal {B}_{UV}$. Moreover, we require that it also preserve the topological junctions between line operators in $\mathcal {B}_{UV}$. In particular, we require the isomorphisms (see Fig. 8)
  B.3 $$\begin{aligned} \Phi _{x,y}: F_{RG}(x) \times F_{RG}(y) \xrightarrow {\sim } F_{RG}(x \times y)~. \end{aligned}$$
  These relations ensure that we have an invertible map relating the fusion spaces of $\mathcal {B}_{UV}$ and their images under $F_{RG}$ in $\mathcal {B}_{IR}$.^56^ We must also demand compatibility with the *F* matrices of both $\mathcal {B}_{UV}$ and $\mathcal {B}_{IR}$ (see Fig. 9).
- Since the braiding of two line operators in $\mathcal {B}_{UV}$ does not depend on the distance between the line operators, the RG flow must preserve the braidings^57^ (see Fig. 10)
  B.4 $$\begin{aligned} R_{x,y} \circ \Phi _{x,y} = \Phi _{y,x} \circ R_{F_{RG}(x),F_{RG}(y)}~. \end{aligned}$$
  By choosing a basis for the fusion spaces, this condition can be written explicitly as in Fig. 10. Comparing the expressions in Fig. 10 (i) and (ii), we get the equation
  B.5 $$\begin{aligned} \sum _{j}\left[ R_{xy}^{z}\right] _{j}^{k}\left[ \Phi _{x,y}^{c}\right] _{{( a,\psi )( b,\chi ) ,\alpha }^{ z,\xi ,j}}=\sum _{\beta }\left[ \Phi _{y,x}^{c}\right] _{( b,\chi )( a,\psi ) ,\beta }^{ z,\xi ,k}\left[ R_{ab}^{c}\right] _{\alpha }^{\beta }~. \end{aligned}$$

Let $p_{UV}(F,R)\in \mathcal {A}^{(1)}(\mathcal {B}_{UV})$ be an invariant of $\mathcal {B}_{UV}$. $p_{UV}(F,R)$ can be interpreted as the value assigned to a network of line operators in $\mathcal {B}_{UV}$. For example, it could be a link invariant for a link colored with the line operators in $\mathcal {B}_{UV}$. The compatibility of $\mathcal {I}_{RG}$ with the fusion and braiding of the two categories $\mathcal {B}_{UV}$ and $\mathcal {B}_{IR}$ implies that the link invariant can be evaluated by first pushing the link through the interface $\mathcal {I}_{RG}$ onto the right IR phase, and evaluating the link invariant in $\mathcal {B}_{IR}$. In other worlds,

B.6 $$\begin{aligned} p_{UV}(F,R)=p_{IR}(F_{RG}(F),F_{RG}(R))~, \end{aligned}$$

which is precisely the anomaly matching condition.

Now, we are ready to prove Theorem 6 from the main text which we rephrase in light of the above discussion:

### Theorem 6

Consider an RG interface, $\mathcal {I}_{RG}$, such that the topological data is mapped via $F_{RG}$ satisfying the assumptions above. Then, we have

(i) $$N_{F_{RG}(x)F_{RG}(y)}^{F_{RG}(z)}=N_{xy}^z$$.
(ii) $$d_{F_{RG}(x)}=d_x$$.
(iii) If $N_{F_{RG}(x)}^a\ne 0$, then $\theta _{a}=\theta _{x}$ .

### Proof

(i) By assumption, $\Phi _{x,y}$ are isomorphisms between fusion spaces $V_{xy}^z$ and $V_{F_{RG}(x)F_{RG}(y)}^{F_{RG}(z)}$. Therefore, we have $N_{F_{RG}(x)F_{RG}(y)}^{F_{RG}(z)}=N_{xy}^z~.$
(ii) Let $K_{UR}$ be the ring formed by the fusion of line operators in $\mathcal {B}_{UV}$. The ring $K_{IR}$ is defined similarly. From (*i*), we know that
  B.7 $$\begin{aligned} F_{RG}: K_{UV} \rightarrow K_{IR}~, \end{aligned}$$
  is a ring homomorphism. A ring homomorphism preserves Frobenius-Perron dimension of line operators [10, Proposition 3.3.13].
  B.8 $$\begin{aligned} \text {FPdim}(F_{RG}(x))=\text {FPdim}(x)~. \end{aligned}$$
  We will assume that both $\mathcal {Q}_{UV}$ and $\mathcal {Q}_{IR}$ are unitary quantum field theories. Therefore, the braided fusion categories $\mathcal {B}_{UV}$ and $\mathcal {B}_{IR}$ are unitary. In a unitary braided fusion category, the Frobenius-Perron dimensions and quantum dimensions agree [10, Proposition 9.5.1]. Therefore,
  B.9 $$\begin{aligned} d_{F_{RG}(x)}=d_{d_x} ~ \forall x \in \mathcal {B}_{UV}~. \end{aligned}$$
  Note that this result is independent of the existence of $\Phi _{x,y}$ and only depends on $F_{RG}$ being a ring homomorphism between $K_{UV}$ and $K_{IR}$.
(iii) Using the fact that $\Phi _{x,y}$ is an isomorphism, we can rewrite (B.5) as
  B.10 $$\begin{aligned} \left[ R_{xy}^{z}\right] _{j}^{k}=\sum _{\alpha , \beta , (a,\psi ), (b,\chi )} \left[ \Phi _{y,x}^{c}\right] _{( b,\chi )( a,\psi ) ,\beta }^{ z,\xi ,k}\left[ R_{ab}^{c}\right] _{\alpha }^{\beta } \left[ (\Phi _{x,y}^c)^{-1} \right] _{z,\xi ,j}^{(a,\psi ),(b,\chi ),\alpha }~. \end{aligned}$$
  Then,
  B.11 $$\begin{aligned} \sum _j \left[ R_{xx}^{z}\right] _{j}^{j}= \sum _{j,\alpha , \beta , (a,\psi ), (b,\chi )} \left[ \Phi _{x,x}^{c}\right] _{( b,\chi )( a,\psi ),\beta }^{ z,\xi ,j}\left[ R_{ab}^{c}\right] _{\alpha }^{\beta } \left[ (\Phi _{x,x}^c)^{-1} \right] _{z,\xi ,j}^{(a,\psi ),(b,\chi ),\alpha }~. \end{aligned}$$
  Note that the LHS is independent of both *c* and $\xi$. Therefore, we have
  B.12 $$\begin{aligned}  &   \sum _{c, \xi } d_c \sum _{j,\alpha , \beta , (a,\psi ), (b,\chi )} \left[ \Phi _{x,x}^{c}\right] _{( b,\chi )( a,\psi ),\beta }^{ z,\xi ,j}\left[ R_{ab}^{c}\right] _{\alpha }^{\beta } \left[ (\Phi _{x,x}^c)^{-1} \right] _{z,\xi ,j}^{(a,\psi ),(b,\chi ),\alpha } \nonumber \\  &   \quad = \sum _{c, \xi } d_c \left[ R_{xx}^{z}\right] _{j}^{j} = d_z \left[ R_{xx}^{z}\right] _{j}^{j}~, \end{aligned}$$
  where, in the last equality, we used $\sum _{c,\xi } d_c = \sum _{c} N_{F_{RG}(z)}^{c} c = d_z$. Rearranging the sums in the equation above, we can write
  B.13 $$\begin{aligned}  &   \sum _{z,j} \left[ R_{xx}^{z}\right] _{j}^{j}\nonumber \\  &   \quad = \sum _c d_c \sum _{\alpha , \beta , (a,\psi ), (b,\chi )} \sum _{z,\xi ,j} \left[ \Phi _{x,x}^{c}\right] _{( b,\chi )( a,\psi ),\beta }^{ z,\xi ,j}\left[ R_{ab}^{c}\right] _{\alpha }^{\beta } \left[ (\Phi _{x,x}^c)^{-1} \right] _{z,\xi ,j}^{(a,\psi ),(b,\chi ),\alpha } \nonumber \\  &   \quad = \sum _c d_c \sum _{\alpha ,\beta ,(a,\psi ),(b,\chi )} \left[ R_{ab}^{c}\right] _{\alpha }^{\beta } \delta _{(a,\psi ),(b,\chi )} \delta _{\alpha ,\beta } \nonumber \\  &   \quad = \sum _c d_c \sum _{\alpha ,(a,\psi )} \left[ R_{aa}^{c}\right] _{\alpha }^{\alpha } = N_{F_{RG}(x)}^a \sum _{c, \alpha } d_c \left[ R_{aa}^{c}\right] _{\alpha }^{\alpha } ~, \end{aligned}$$
  where, in the second equality, we used the invertibility of $\Phi _{x,x}^c$. Therefore, using $\theta _x =\frac{1}{d_x} \sum _{z,j} \left[ R_{xx}^x \right] _j^j$ and $\theta _a =\frac{1}{d_a} \sum _{c,\alpha } \left[ R_{aa}^c \right] _\alpha ^\alpha$, we get
  B.14 $$\begin{aligned} d_x \theta _x = \sum _{a} N_{F_{RG}(x)}^a d_a \theta _a~. \end{aligned}$$
  Regarranging the terms, we get
  B.15 $$\begin{aligned} \sum _a \left( N_{F_{RG}(x)}^a \frac{d_a}{d_x} \right) \frac{\theta _a}{\theta _x} =1~. \end{aligned}$$
  The sum is over phases with real non-negative coefficients. Moreover, $\sum _a N_{F_{RG}(x)}^a \frac{d_a}{d_x} = 1$. Therefore, we have
  B.16 $$\begin{aligned} \theta _a = \theta _x ~ \forall ~ a \text { such that } N_{F_{RG}(x)}^a\ne 0~. \end{aligned}$$

$$\square $$

### Corollary

The braiding of line operators in $\mathcal {B}_{UV}$ and $\mathcal {B}_{IR}$ are related as follows

B.17 $$\begin{aligned} S_{xy} = \sum _{a,b} N_{F_{RG}(x)}^a N_{F_{RG}(y)}^b~ S_{ab}~. \end{aligned}$$

### Proof

We have

B.18 $$\begin{aligned} S_{x,y}= \sum _{z} N_{xy}^z \frac{\theta _z}{\theta _x \theta _y} d_z~. \end{aligned}$$

Using Theorem 6, we find that

B.19 $$\begin{aligned} \theta _x \theta _y S_{x,y}= &   \sum _{z} N_{xy}^z \theta _z d_z = \sum _{z} N_{xy}^z \sum _{c} N_{F_{RG}(z)}^c d_c \theta _c \end{aligned}$$

B.20 $$\begin{aligned}= &   \sum _{a,b,c} N_{F_{RG}(x)}^a N_{F_{RG}(y)}^b N_{ab}^c d_c \theta _c = \sum _{a,b} N_{F_{RG}(x)}^a N_{F_{RG}(y)}^b\theta _a \theta _b S_{a,b}~,\qquad \end{aligned}$$

where, in the third equality, we used the isomorphism between $F_{RG}(x) \times F_{RG}(y)$ and $F_{RG}(x\times y)$. Using $\theta _a = \theta _x$ for all a such that $N_{F_{RG}(x)}^a\ne 0$ and $\theta _b = \theta _y$ for all a such that $N_{F_{RG}(y)}^b\ne 0$, we obtain

B.21 $$\begin{aligned} S_{x,y} = \sum _{a,b} N_{F_{RG}(x)}^a N_{F_{RG}(y)}^b S_{a,b} ~. \end{aligned}$$

$$\square $$

As a final point, note that, Theorem 6 is necessary but not sufficient for $F_{RG}: \mathcal {B}_{UV} \rightarrow \mathcal {B}_{IR}$ to be a braided monoidal functor. In particular, note the following two obstructions:

- The first two properties of Theorem 6 show that $F_{RG}: \mathcal {K}_{UV} \rightarrow \mathcal {K}_{IR}$ is a ring homomorphism between *UV* and *IR* fusion rings [10]. However, this condition is not enough to give a lift to a monoidal functor between tensor categories, $\mathcal {B}_{UV} \rightarrow \mathcal {B}_{IR}$.
- If we bypass the first obstruction by demanding the existence of a monoidal functor from $\mathcal {B}_{UV}\rightarrow \mathcal {B}_{IR}$ obeying all the properties of Theorem 6, we still cannot specify the *R*-matrix if $\mathcal {B}_{UV}$ and $\mathcal {B}_{IR}$ are degenerate [100]. We would need to specify the extra data of the embedding to the Drinfeld center ($\mathcal {B}_{UV}\rightarrow \mathcal {Z}(\mathcal {B}_{UV})$ and $\mathcal {B}_{IR}\rightarrow \mathcal {Z}(\mathcal {B}_{IR})$) which then allows us to fix the *R*-matrices in terms of the data of Theorem 6 and the generalized second Frobenius-Schur indicators [100].

## Footnotes

1 Although the original definition of one-form symmetry [9] involved invertible symmetries, we define one-form symmetry to also cover the case of a non-invertible categorical symmetry generated (in $2+1$d) by topological lines.

2 Since these lines have real self-statistics, this question amounts, in some sense, to classifying “non-Anyonic” symmetries. However, this notion is somewhat misleading because of the possibility of non-trivial (but, as we will see, still real) mutual statistics (e.g., consider Kitaev’s toric code).

3 We define a non-invertible 1-form symmetry, $\mathcal {B}$, to be non-intrinsically non-invertible if there is a topological manipulation, $\rho$, such that $\rho (\mathcal {B})$ only contains invertible genuine line operators (note that, throughout this work, when we refer to line operators, we have in mind genuine lines). This definition is closely related to the notion of non-intrinsically non-invertible symmetry in 1+1d QFTs studied in [11–13]. However, note that in 1+1d, this definition is equivalent to a fusion category being group-theoretical in the sense of [10] (see also [13]). This statement is no longer true in 2+1d. For example, $\text {Ising} \boxtimes \overline{\text {Ising}}$ is non-intrinsically non-invertible in 2+1d because it can be obtained form gauging the $\mathbb {Z}_2$ electromagnetic duality symmetry of the 2+1d $\mathbb {Z}_2$ Dijkgraaf–Witten theory. However, $\text {Ising} \boxtimes \overline{\text {Ising}}$ is not group-theoretical.

4 Interestingly, two of the present authors recently showed that non-invertible zero-form symmetries in $2+1$d generated by condensing lines are also non-intrinsically non-invertible [14]. In that context, non-invertibility arises from the presence of non-trivial bosons that can be condensed in all of spacetime. Below, we will see that such bosons are also necessary (though, unlike in the zero-form case, not sufficient) to realize non-invertible BFB categories.

5 Metaplectic modular categories are closely related to the categories we consider here in the sense that they are also related to Chern-Simons theories with $\textrm{Spin}(N)$ gauge groups (although with *N* odd).

6 When the *S*-matrix is non-degenerate, it is unitary up to a normalization factor, $D:=\sqrt{\sum _{\ell _i} d_{\ell _i}^2}$.

7 Since we will mostly focus on unitary theories, the quantum dimensions are just the categorical Frobenius-Perron dimensions,  More physically, we can think of the quantum dimensions as $S^3$ expectation values of loops.

8 Note that for the *S* matrix we are using the normalization in (2.1).

9 A prime MTC is an MTC that cannot be written as the (Deligne) product of two or more other MTCs. Any Abelian MTC, $\mathcal {M}$, can be written in terms of a (not always unique) prime factorization  where the $\mathcal {M}_i$ are prime MTCs. Each factor in the above decomposition is closed under fusion, and each $\mathcal {M}_i$ braids trivially with any $\mathcal {M}_j$ labeled by $j\ne i$.

10 One can look at the full set of rank-three MTCs (i.e., MTCs with three simple objects) in [22] to see this theory is inconsistent. Another way to argue that this theory is inconsistent is to note that the quantum dimensions of such a theory would live in an extension of the rationals, $\mathbb {Q}(\sqrt{2})$ (since $d_{\sigma }=\sqrt{2}$). In an MTC, this extension is determined by the conductor, which is the smallest $N>0$ such that $\theta (\ell _i)^N=1$ for all $\ell _i$. In particular, we should have $d_{\ell _i}\in \mathbb {Q}(\xi _N)$ for some primitive *N*th root of unity, $\xi _N$ [23]. However, $\sqrt{2}\not \in \mathbb {Q}(\xi _2)\cong \mathbb {Q}$.

11 This same fact is behind the appearance of the FS indicator in the quantum mechanical addition of spins [24, 25].

12 The FS indicator can be defined for line operators in any unitary fusion category. Indeed, if a line operator, $\ell$, is non-self-dual, then $\nu _2(\ell ):=0$.

13 Note that, from the perspective of higher gauging [32], the difference between the FS indicator and the correction term in (2.19) can be understood in terms of 2-gauging. Indeed, up to normalization, this difference arises from 2-gauging $J:=\sum _{\ell } d_{\ell } ~\ell$ on a cycle surrounding $\ell _i \times \ell _i$. This maneuver projects onto $\mathcal {Z}_{M}^\textrm{bos}(\mathcal {B})$.

14 Another way to see this is to use the fact that all (pseudo-unitary) super-MTCs admit a minimal modular extension [33]. The modular extension is a $\mathbb {Z}_2$-graded category, $\mathcal {M}=\mathcal {M}_0 + \mathcal {M}_1$, where $\mathcal {M}_0=\mathcal {B}$. The modular *S* matrix of $\mathcal {M}$ is given by (2.1). Therefore, it is clear that $S|_{\mathcal {M}_0}$ is real. Now, from [35, Theorem 3.5] we have $S|_{\mathcal {M}_0}= \hat{S} \otimes S_{\text {SVec}}$, where $S_{\text {SVec}}$ is the *S* matrix of SVec. Furthermore, [36, Proposition 2.7] shows that $\hat{S} \overline{\hat{S}}=\frac{D^2}{2} I$, and $\hat{S}^2 = \frac{D^2}{2} C$, where *C* is the charge conjugtion matrix. Since $\hat{S}$ is real in our case, $C=I$. Therefore, all lines in a super-MTC with real spins are self-dual.

15 This statement is to be contrasted with non-trivial unitary symmetries of a non-Abelian TQFT which can act trivially on all line operators [38].

16 It is of course also interesting to think instead in terms of UV completions via lattice models, but we will not do so here.

17 Closely related quiver theories appear in the context of dimensional deconstruction [41] (see also [42] for a more closely related study in $2+1$d where circular quivers are related to a fourth space-time dimension). It would be interesting to understand if there are consequences for $3+1$d physics.

18 Indeed, we have already encountered such examples in the non-topological QFTs of (2.25) and (2.26). Note that the case $\mathcal {Z}_M(\mathcal {B})\cong \textrm{Vec}$ corresponds to $\mathcal {B}$ being an MTC, while $\mathcal {Z}_M(\mathcal {B})\cong \textrm{SVec}$ corresponds to $\mathcal {B}$ being a super-MTC.

19 The fact that *G* is unique follows from Tannaka-Krein reconstruction applied to $\mathcal {Z}_M^\textrm{bos}(\mathcal {B})$.

20 This statement can be easily seen by invoking [45, Theorem 3.1] and noting that Morita equivalence is an equivalence of Drinfeld centers. Then, we can invoke the relation $F_2\boxtimes F_2\cong E_2\boxtimes E_2$.

21 We can always achieve this situation for any *G* as follows: by Cayley’s theorem, there exists a positive integer *N* such that $G\subset S_N$. Now, consider a theory with *N* free scalar fields (real or complex). The $S_N$ symmetry acts on these free scalars faithfully as a permutation representation and so too does the subgroup *G*. Now, using a classic theorem by Burnside, tensor powers of a faithful representation of a compact group contain all of its irreducible representations. This result ensures that OPEs of the operators we have constructed contain local operators in all irreps of the group *G*.

22 See the physical discussion in [51, 52] for more details. Technically speaking, $\beta$ need not vanish if we are willing to gauge the one-form symmetry as well (i.e., gauge the corresponding 2-group).

23 A complete discussion from the perspective of 3d TQFTs and category theory can be found in [51, 54].

24 This symmetry action can be extended consistently to at least one minimal modular extension of $\mathcal {M}$ (in particular, to $\widetilde{\mathcal {M}} := D(\mathbb {Z}_{2})\boxtimes D(\mathbb {Z}_{2})$).

25 Or, from a simpler though more mathematical perspective, we can obtain any BFB by equivariantizing the Abelian (super-) MTCs in question with respect to *G*.

26 In more general cases, we can think of $\mathcal {B}_{UV}$ as being a premodular fusion subcategory of a larger (possibly non-semisimple) UV one-form symmetry category, $\mathcal {C}_{UV}$.

27 Rendering a genuine line non-genuine changes the topology of the line, since it must be attached to a surface.

28 In $1+1$d, when the gapped IR phase is trivial, we have $\mathcal {B}_{IR}\cong \text {Vec}$ making the RG functor, $F_{RG}$, a fiber functor. Constraints on $1+1$d RG flows from the existence of fiber functors were studied in [58–60]. RG functors for RG flows from $1+1$d CFTs to possibly non-trivial IR gapped or gapless phase were studied in [61–64]. In $3+1$d, RG functors for BPS line defects were studied in [65].

29 $F_{RG}(\mathcal {B}_{UV})$ is closed because otherwise, as we go back up the RG flow from the IR to the UV, a product of two topological lines in $F_{RG}(\mathcal {B}_{UV})$ would produce non-topological lines.

30 Although conceptually simple, implementing the RG interface in practice is typically non-trivial (e.g., see [66–69]).

31 By definition, the *F* and *R* matrices of a braided fusion category form a complete set of invariants that determine the braided fusion category. However, note that $\mathcal {A}^{(1)}(\mathcal {B})$ is a collection of basis-independent combinations of the *F* and *R* matrices. Indeed, by [70, 71] (see also [72]), a finite number of such invariants exist for multiplicity-free braided fusion categories. We assume that this statement holds in general.

32 Formally, the RG interface, $\mathcal {I}_{RG}$, describes how all local and extended operators of the UV QFT, $\mathcal {Q}_{UV}$, map to those in the IR QFT, $\mathcal {Q}_{IR}$. For example, $\mathcal {I}_{RG}$ specifies how topological surface and line operators in the UV are mapped to their counterparts in the IR. The topological surfaces and lines of a 2+1d QFT form a 2-category. Therefore, studying RG flows in this general setting requires understanding monoidal 2-functors between two 2-categories. We leave this study for future work and instead focus on the topological lines and corresponding 1-categories.

33 We note that this theorem is a necessary but not sufficient condition for having a braided monoidal RG functor. See Appendix B for more details.

34 In 1+1d, obstructions to gapped IR phases from quantum dimensions of UV line operators were studied in [58–60].

35 Recall that $\mathcal {Z}_M^\textrm{bos}(F_{RG}(\mathcal {B}_{UV}))$ is the subcategory of bosonic line operators that are transparent in $F_{RG}(\mathcal {B}_{UV})$. This subcategory must be the same as $F_{RG}(\mathcal {Z}^\textrm{bos}_M(\mathcal {B}_{UV}))$. Indeed, any $\ell \in \mathcal {Z}_M^\textrm{bos}(F_{RG}(\mathcal {B}_{UV}))$ must come from lines in $F_{RG}(\mathcal {Z}^\textrm{bos}_M(\mathcal {B}_{UV}))$. Otherwise, when we go back up the RG flow to the UV, $\ell$ is mapped to lines that have non-trivial ’t Hooft anomalies, which is inconsistent. Moreover, by ’t Hooft anomaly matching, any $\ell \in F_{RG}(\mathcal {Z}^\textrm{bos}_M(\mathcal {B}_{UV}))$ must braid trivially with $F_{RG}(\mathcal {B}_{UV})$ and hence $\ell$ must be a member of $\mathcal {Z}_M^\textrm{bos}(F_{RG}(\mathcal {B}_{UV}))$.

36 In particular, since Theorem 6 corresponds to the matching of modular data, we see that it is necessary but not in general sufficient to match anomalies.

37 More generally, $\mathcal {B}_{UV}$ may contain a closed BFB subcategory, $\tilde{\mathcal {B}}_{UV}$. It is straightforward to modify our argument for this case by focusing on $\tilde{\mathcal {B}}_{UV}$ instead.

38 For invertible 1-form symmetries, ’t Hooft anomaly can be cancelled using a 3+1d invertible TQFT (an invertible 1-form Symmetry Protected Topological Phase) [9, 75–77]. In this case, the invariance of the anomaly under the RG flow is a consequence of the fact that the 3+1d theory is a TQFT. Theorem 7 applies to a general (non-invertible) 1-form symmetry and the RG invariant TQFT $\mathcal {M}$ is a 2+1d TQFT. It will be interesting to understand the anomaly inflow picture for (non-invertible) 1-form symmetries.

39 Note that a braided monoidal functor from a (super)-MTC to a premodular category is injective/fully-faithful, implying that $\mathcal {M}$ is preserved along the RG flow.

40 A formal argument for this result uses the fact that $F_{RG}: \mathcal {B}_{UV} \rightarrow \mathcal {B}_{IR}$ is a braided monoidal functor. In particular, it is a braided monoidal functor from Rep$(G^{UV})$ to $\mathcal {B}_{IR}$. Any such functor is an embedding of Rep(*H*) for some subgroup $H\le G$ in $\mathcal {B}_{IR}$ (see, for example, [78, Section 3.3.1]). In our case, *N* is the largest subgroup of $G^{UV}$ such that the restrictions of all representations in Rep$(G^{UV}/N)$ to *N* result in (several copies of) the trivial representation of *N*. Indeed, since $F_{RG}$ trivializes the line operators in $\mathcal {C}_\textrm{triv}$, it is the restriction map from irreducible representation of $G^{UV}$ to *N*.

41 We can further justify (3.14) as follows. It is always possible to equivariantize with respect to a discrete *G* symmetry by first equivariantizing with respect to a normal sugroup, $H\lhd G$, and then equivariantizing with respect to *G*/*H* (e.g., see the discussion in [51]). Proceeding in this way, first equivariantizing (3.11) with respect to $F_{RG}(G^{UV})$ gives $F_{RG}(\mathcal {B}_{UV})$. Further equivariantizing with respect to $G^{UV}/F_{RG}(G^{UV})$ gives $\mathcal {B}_{UV}$. As a result, (3.14) follows from condensation.

42 Note that this statement, with its implicit ordering, is intuitively consistent with the *F*-theorem [80, 81], since topological degrees of freedom contribute to this quantity.

43 However, the examples of RG flows that we consider in this work do not include examples of the partial trivializations in (3.15). It will be interesting to find examples of this potential phenomenon, but we leave this point for future work.

44 In the case with transparent fermions, we should consider the more complicated obstruction theory touched upon in the example at the end of Sect. 2.3.

45 When $\mathcal {M}_{IR}$ is gapped, it is a $\mathcal {B}_{UV}$-symmetric 2+1d TQFT.

46 In certain cases, this logic can be rephrased in terms of the methods described in [85].

47 For example, as in the theories discussed in [86].

48 Gauge invariance requires $\ell ^{\times n}$ to contain the identity line. In other words, the *n*-fold product of $\ell$ satisfies

49 More precisely, we have $\mathcal {M}_{IR}/F_{RG}(\mathcal {B}_{UV})\cong \textrm{SPT}(\mathbb {Z}_2)_{\omega _3}$.

50 In this analysis, $\epsilon$ and $\overline{\epsilon }$ are the fermionic lines in $\textrm{Ising}$ and $\overline{\textrm{Ising}}$ respectively.

51 A different argument was used in [85] for the cases of interest there.

52 A minimal modular extension would be of the form $SU(2)_1\boxtimes D_{\omega }(\mathbb {Z}_2\times \mathbb {Z}_2)$, where the second factor is a (twisted) Dijkgraaf–Witten theory.

53 It would also be interesting to understand if there is any relation between the simplicity of classifying metaplectic modular categories and BFB categories. One common feature is the close relation with CS theories having $\textrm{Spin}(N)$ gauge group (although in the metaplectic case, *N* is odd).
